# Coastal groundwater phosphorus drives global acceleration of algal blooms

**DOI:** 10.1038/s41467-026-75420-y

**Published:** 2026-07-16

**Authors:** K. H. Cheng, Joseph H. W. Lee, Jiu Jimmy Jiao, Adina Paytan, Donald M. Anderson, Weijun Cai, Holly A. Michael, Mathew A. Charette, Shuh-Ji Kao, Jin Wu, Xin Luo

**Affiliations:** 1 School of Life Sciences, The Chinese University of Hong Kong; Shatin, Hong Kong, China; 2 Department of Earth and Planetary Sciences, The University of Hong Kong; Pokfulam, Hong Kong, China; 3 School of Biological Sciences, The University of Hong Kong; Pokfulam, Hong Kong, China; 4 Key Lab of River Basin Digital Twinning, Ministry of Water Resources, Faculty of Innovation Engineering, Macau University of Science and Technology, Macau, China; 5 The University of Hong Kong, Shenzhen Institution of Research and Innovation (SIRI), Shenzhen, China; 6 Department of Earth and Planetary Sciences, University of California at Santa Cruz, Santa Cruz, CA USA; 7 Biology Department, Woods Hole Oceanographic Institution, Woods Hole, MA USA; 8 Department of Geological Sciences, University of Delaware, Newark, DE USA; 9 Woods Hole Oceanographic Institution, Woods Hole, MA USA; 10 State Key Laboratory of Marine Resources Utilization in South China Sea, Hainan University, Haikou, China; 11 Institute for Climate and Carbon Neutrality, The University of Hong Kong; Pokfulam, Hong Kong, China; 12 State Key Laboratory of Agrobiotechnology, The Chinese University of Hong Kong; Shatin, Hong Kong, China; 13 Sea Space Agent Laboratory, Department of Earth and Planetary Sciences, The University of Hong Kong; Pokfulam, Hong Kong, China

**Keywords:** Hydrogeology, Element cycles, Hydrology, Marine biology

## Abstract

Coastal algal blooms (CABs) are accelerating worldwide. This intensification is particularly puzzling in regions where nitrogen inputs have been curbed, yet blooms persist or worsen. Here we reveal excess dissolved inorganic phosphorus (DIP) from coastal groundwater as a critical, previously overlooked driver. Combining a process-based physical-chemical framework with global datasets, we find decadal CAB trends are more strongly linked to coastal DIP than to nitrogen availability or water-column stability. A global meta-analysis with site-specific time series demonstrates anoxic groundwater releases substantial DIP. This mechanism explains continued CABs under nitrogen control and aligns with a shift toward phosphorus limitation in many coastal regions. Spatial and temporal variation in CAB trends reflects aquifer geochemistry, with reducing groundwater fueling blooms and oxidized systems suppressing them. Our findings establish groundwater as a missing link in the modern coastal nutrient cycle and underscore the urgency for integrated nitrogenphosphorus management to mitigate the escalating impacts of CABs.

## Abstract (web summary)

Phosphorus-rich inputs from anoxic groundwater emerge as a key driver of accelerating global coastal algal blooms, offering an explanation for why these blooms persist despite strict nitrogen controls.

## Introduction

Coastal algal blooms (CABs), episodes of anomalously high phytoplankton biomass, have emerged as a major and escalating threat to marine ecosystems, coastal economies, and human health^1,2^. Harmful CABs can cause mass fish kills, beach closures, shellfish toxicity, and large-scale hypoxia, undermining fisheries, aquaculture, and public health systems^2–4^. The economic toll is substantial, with global losses estimated at up to US$2 billion annually^5^, reflecting both direct damages and lost ecosystem services. As coastal populations expand and pressures from resource use intensify^6,7^, there is an urgent need to understand and mitigate CABs, not only to safeguard ecosystem resilience but also to protect livelihoods in a changing climate.

Over the past two decades, long-term monitoring of CABs using chlorophyll*a* concentrations (Chl-*a*; Supplementary Fig. 1a), cell counts^3,4^ or proxy indices such as fluorescence line height^1^ has revealed a striking increase in CAB frequency and intensity across diverse coastlines^1,2,4,8^. This acceleration presents a critical ecological paradox: blooms persist or even worsen in regions where nitrogen inputs have been effectively reduced through decades of environmental regulation^9,10^. Resolving this paradox demands a re-examination of the dominant drivers of contemporary bloom expansion on a global scale. Two broad, non-exclusive categories of drivers are hypothesized, yet their relative global importance remains contested. First, physical drivers linked to anthropogenic climate change factors, including ocean warming, altered wind regimes, and shifting precipitation patterns^7,11,12^, are often presumed to enhance water column stratification, creating conditions favorable for phytoplankton accumulation. Intensified coastal upwelling^1,11,12^ has also been proposed, yet its strongest influence occurs near the shelf break, whereas most reported CABs are concentrated in nearshore and estuarine zones^2,13^. Such spatial mismatches suggest that additional, potentially under-recognized mechanisms are at play. Second, chemical drivers associated with localized human activities, such as coastal infrastructure development and aquaculture expansion^1,6,11,12^, can modify hydrodynamics and nutrient cycling, altering the flux, form, and stoichiometry of nutrient input^14,15^. While anthropogenic nitrogen (N) and phosphorus (P) loads have been heavily managed, shifts in the relative importance of different nutrient sources and their chemical forms are less well understood. Specifically, a key unresolved question is whether an under-appreciated nutrient source delivering excess dissolved inorganic phosphorus (DIP) relative to N could act as a catalytic trigger for blooms in coastal waters transitioning toward P limitation.

Progress on this question is hindered by fragmented prior research. Many studies focus on regional phenomena or single driver categories, leaving a lack of  a synthesized, multi-decadal global integrated perspective that jointly evaluates physical and chemical controls. Moreover, if excess DIP is indeed the prevailing signal, its provenance must be traced. As classical end-members possess fundamental limitations in both physical flux and stoichiometric signatures (often N:P > 16:1), we must investigate benthic and subterranean pathways. Submarine groundwater discharge (SGD)^3,4^ emerges as a compelling candidate, as coastal aquifers possess unique geochemical conditions capable of concurrently mobilizing phosphorus and removing nitrogen^16,17^, thereby generating low N:P fluxes.

**Fig. 1:**
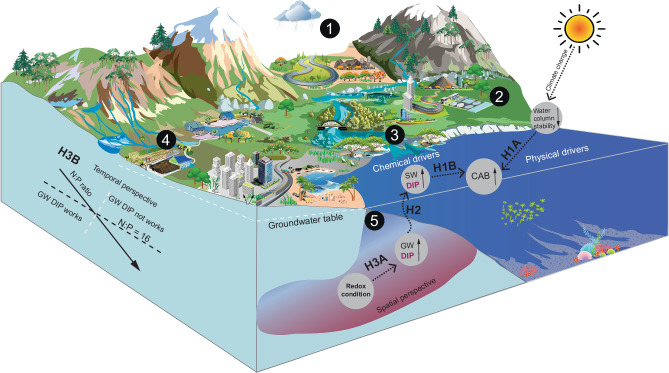
Sequential conceptual framework and hypotheses testing the drivers of accelerated global coastal algal blooms (CABs). The central block diagram illustrates a multi-scale analytical framework designed to evaluate the physical and chemical forcing mechanisms driving the global acceleration of CABs (indicated by the upward arrow in CAB). Testing physical vs. chemical drivers (H1A vs. H1B): H1A (physical drivers) tests whether climate change acts as the dominant driver by increasing water-column stability (e.g., through enhanced thermal stratification), which creates a physical environment favoring bloom development. H1B (chemical drivers) tests whether chemical shifts in nutrient stoichiometry, specifically an increase in seawater (SW) dissolved inorganic phosphorus (DIP), acts as the primary forcing mechanism sustaining and accelerating blooms. Identifying nutrient pathways and sources (Pathways 1–5 and H2): Circled numbers indicate five potential pathways delivering nutrient fluxes to the coastal ocean: (1) atmospheric deposition, (2) sewage effluent, (3) riverine discharge, (4) agricultural runoff, and (5) coastal groundwater (GW) discharge. H2 (groundwater source) proposes that GW serves as the predominant ubiquitous pathway delivering excess DIP directly to coastal waters (SW). Spatiotemporal modulations of groundwater impact (H3A and H3B): H3A (spatial) evaluates how spatial heterogeneity in global CAB trends is regulated by the pre-existing redox states of local coastal aquifers. H3B (temporal) explains how the catalytic influence of groundwater-derived DIP varies temporally based on coastal nutrient limitation regimes.

Here, rather than attempting to falsify mechanisms that may have local significance, we employ a sequential analytical framework to quantitatively evaluate the predominant macro-scale drivers of accelerating CABs and trace their origins. We test a structured set of hypotheses to resolve the paradox surrounding this global phenomenon (Fig. 1). First, using a spatially explicit, multi-decadal (1998–2022) framework integrating coastal Chl-*a* records with physical and chemical parameters, we assess the relative global dominance of two major mechanisms: climate-driven increases in water-column stability (H1A), versus chemical shifts in nutrient stoichiometry (H1B), particularly elevated DIP relative to DIN. Second, upon identifying a widespread signal of excess DIP, we investigate the proposition (H2) that this DIP originates substantially from coastal groundwater, a pathway uniquely equipped to deliver low N:P ratio inputs due to coupled biogeochemical processes (P desorption and denitrification). Finally, to account for the striking spatial and temporal variability of blooms, we evaluate an integrated mechanism: how spatial heterogeneity in CAB trends reflects the pre-existing redox state of local aquifers (H3A, the spatial switch), and how the catalytic influence of groundwater DIP is governed by a dual temporal dynamic involving short-term redox fluctuations and long-term shifts in coastal nutrient limitation regimes (H3B). By systematically navigating this framework, our study isolates excess DIP as the predominant driver of recent CAB acceleration, traces its source to the ubiquitous yet overlooked coastal groundwater pathway, and establishes a macro-scale mechanism linking aquifer redox geochemistry to coastal bloom vulnerability. Ultimately, this work highlights that future human activities altering groundwater chemistry could inadvertently expand regions susceptible to phosphorus-driven blooms, underscoring a critical frontier for global coastal management.

## Results and discussion

### Evaluating physical stability versus excess DIP as the primary macro-scale driver

**Fig. 2:**
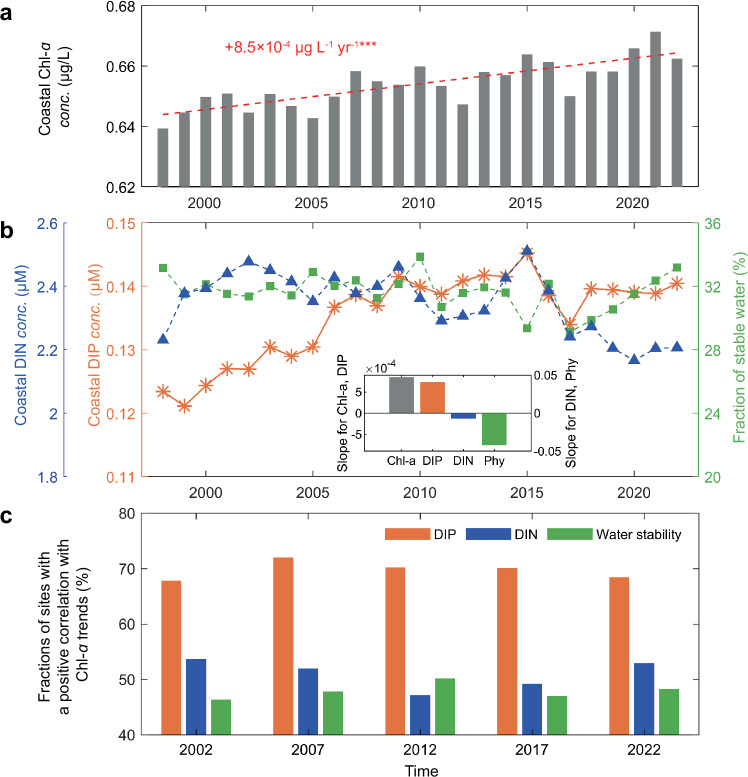
Chemical drivers of coastal phosphorus availability accelerate global coastal algal blooms more than physical controls. **a** Interannual variability of mean coastal chlorophyll-*a* (Chl-*a*) concentrations (µg L^−1^) from 1998 to 2022. The red dashed line denotes the long-term linear regression trend, with the calculated annual growth rate indicated (two-sided *t*-test, ***p < 0.001). **b** Long-term temporal trajectories of chemical and physical drivers in global coastal zones, including coastal dissolved inorganic nitrogen (DIN, blue triangles and a dashed line, far-left axis), dissolved inorganic phosphorus (DIP, orange asterisks and a dashed line, middle-left axis), and the fraction of stable water sites (Phy, green squares and dashed line, right axis; serving as a proxy for physical forcing). The inset bar chart compares the annual change rates (linear regression slopes) for Chl-*a* and DIP (left y-axis, ×10^−4^), alongside DIN and physical stability (Phy, right y-axis). **c** Fractions of sites with synergistic DIP, DIN and Chl-*a* trends, as well as with synergistic water stability and Chl-*a* trend for five different time spans (1998-2002, 1998-2007, 1998-2012, 1998-2017, and 1998-2022) for coastal DIP (orange), DIN (blue), and water column stability (green).

To uncover the central causes behind the recent acceleration of CABs, we examine global trends in Chl-*a*, a widely used proxy for phytoplankton biomass^3,4^, over the past two decades. Our analysis focuses on nearshore waters shallower than 30 m, where blooms most directly affect fisheries, aquaculture, and public health^18^, which  is consistent with the U.S. Environmental Protection Agency’s classification^19^. Across the globe, Chl-*a* concentrations have risen consistently from 1998 to 2022 (Fig. 2a and Supplementary Fig. 1a), confirming that bloom expansion is now a pervasive feature of the coastal ocean^1,20^.

We then ask whether this global acceleration can be explained by broad changes in physical ocean structure or by shifts in nutrient supply. Physical hypotheses often center on water column stability^4,15^, as more stratified conditions generally favor phytoplankton accumulation^21^. To assess changes in water column stability, we employed an integrated vertical mixing index (*E/E*_*c*_) derived from temperature, wind, water clarity, and salinity (Methods). Our analysis reveals a robust shift toward a more mixed state, demonstrated consistently across both spatial frequency and temporal magnitude. Spatially, the global fraction of stable sites (*E/E*_*c*_ < 1) has significantly declined over time (–4.2 × 10⁻² % yr⁻¹, *p* < 0.001; Fig. 2b). Temporally, the long-term trend of the spatially averaged log_10_(*E/E*_*c*_) is positive (where >0 indicates a predominantly mixed state) and continuously increasing (Supplementary Fig. 2b), reflecting a strengthening of average mixing intensity. These two perspectives are physically consistent and converge on the same conclusion: over recent decades, coastal water columns have become progressively less stable and more mixed. Notably, against this background of physical restructuring, global Chl‑*a* trends exhibit only a weak, negative association with water stability (r = –0.07, *p* < 0.001; Supplementary Fig. 3a).

In other words, while large‑scale physical changes have generally made conditions less favorable for blooms, CABs have nevertheless intensified. This mismatch suggests that, at the global aggregate scale, water column stability is unlikely to act as the primary driver of the observed acceleration. In contrast, the chemical signal is striking. DIP has increased significantly across global coastal waters (7.3 × 10⁻⁴ µM yr⁻¹, *p* < 0.001; Fig. 2b), while DIN has declined (–7.4 × 10⁻³ µM yr⁻¹, *p* < 0.001; Fig. 2b), leading to a substantial drop in N:P ratios (–0.15 yr⁻¹, *p* < 0.001; Supplementary Fig. 2a). Annual Chl-*a* concentrations correlate strongly and positively with DIP (*r* = 0.74, *p* < 0.001), and inversely with N:P ratios (*r* = –0.70, *p* < 0.001), far exceeding correlations with DIN (*r* = –0.28, *p* < 0.001) and physical stability (*r* = –0.07, *p* < 0.001) (Supplementary Fig. 3). These relationships are robust across different coastal systems (Supplementary Fig. 4), indicating that phosphorus availability, rather than nitrogen or stratification, is the predominant factor linked to the global rise in CABs.

To further test driver importance more rigorously, we assessed co‑occurring trends between Chl‑*a* and each potential driver across 237,540 coastal sites over multiple cumulative time spans: 1998–2002, 1998–2012, 1998–2017, and 1998–2022 (Methods). At all timescales, DIP–Chl‑a co‑occurrence (67.8–72.1%) was consistently higher than for DIN (47.2–53.7%) or stability (46.4–50.2%), with more than two‑thirds of sites showing matching positive trends in DIP and Chl‑*a* (Fig. 2c). Collectively, this spatially and temporally persistent pattern points to a process delivering excess DIP relative to DIN, a chemical fingerprint that is widespread and sustained, supporting H1B as the primary mechanism driving these coastal dynamics.

Coastal nutrient levels and N:P ratios result from multiple sources and sinks, with phytoplankton uptake as the main removal process. Thus, linking these parameters to Chl-*a* requires accounting for both initial nutrient inputs and their subsequent uptake by phytoplankton. The observed trends, namely rising DIP, declining DIN and N:P ratios, are best explained by a conceptual model of low N:P ratio nutrient inputs (Model 1, N:P < 16:1, Supplementary Fig. 5a). In this scenario, a relative surplus of DIP alleviates phosphorus limitation, enabling phytoplankton to consume available DIN more completely, thereby fueling blooms and leaving residual DIP in the water column (Fig. 2b). Collectively, this geographically widespread source of excess DIP acts as the primary chemical driver of recent CAB acceleration, particularly along densely populated coasts of Asia, Australia, and South America (Supplementary Fig. 1b). However, our spatial analysis reveals distinct geographic and hydrodynamic settings where alternative mechanisms govern Chl-*a* responses (Supplementary Fig. 1c, d). Specifically, while DIP dominance aligns with widespread groundwater inputs, high water column stability becomes the primary control in semi-enclosed, physically stratified regions (e.g., the Mediterranean Sea, the Gulf of Mexico), and distinct DIN dynamics with high N:P inputs (Model 2, N:P > 16: 1, Supplementary Fig. 5b) dictate responses in localized areas of Europe and the eastern United States. This geographic delineation clarifies that while localized physical stratification or specific nitrogen regimes regulate a subset of systems, the overarching global expansion of CABs is overwhelmingly driven by widespread DIP enrichment.

### Tracing the source of excess DIP to coastal groundwater

While our global analysis reveals that excess DIP input relative to DIN is the dominant driver for recent acceleration of CABs, the origin of this phosphorus surplus remains unclear. Coastal waters receive nutrients from a diverse array of sources, including rivers^22,23^, sewage discharge^24^, sediment diffusion^25,26^, atmospheric deposition^27^, and groundwater inputs^28,29^. However, classical endmembers, namely rivers, the atmosphere, and sedimentary diffusion, possess fundamental limitations in both physical flux and elemental stoichiometry that preclude them as the primary drivers of the global DIP surplus. Physically, atmospheric DIP flux is generally negligible due to the inherent lack of a stable gaseous phase in the global phosphorus cycle and the restricted air-sea interface area of shallow (<30 m) coastal zones^26,27^. Rivers act merely as localized “point sources” prone to rapid estuarine dilution^30,31^, with extensive upstream damming further curtailing downstream phosphorus delivery^30–34^. Meanwhile, sedimentary diffusion is profoundly restricted by the physicochemical scavenging of iron oxides (Fe-oxides) within the oxic sediment-water interface, rendering it too localized to explain a coherent global DIP signal^35–37^. Furthermore, beyond these physical constraints, the stoichiometric signatures of these classical sources fundamentally contradict a surplus DIP input. Most terrestrial and atmospheric inputs are intrinsically nitrogen-rich. For example, global riverine N:P ratios have consistently exceeded the Redfield ratio (16:1) in recent decades^32–34^, atmospheric inputs are likewise heavily skewed toward nitrogen^38^, and treated sewage typically emerges DIN-enriched despite raw wastewater being phosphorus-rich^39^.

Coastal groundwater, mediated by saline SGD (recirculated seawater) and porewater exchange^40–42^, presents a different profile. Numerous local and regional studies have identified coastal aquifers, particularly those under anoxic, reducing conditions, as potentially significant sources of nutrient fluxes^41,43,44^. In these systems, phosphate can be released from sediments through phosphate desorption^45,46^, while nitrogen is removed via denitrification^29^, thereby producing low N:P ratios. Moreover, as a massive “non-point source,” the SGD-derived DIP input to the nearshore waters could fundamentally dwarf atmospheric and diffusive fluxes, often rivalling or even exceeding riverine inputs^29,47,48^. This combination makes groundwater a plausible, yet underappreciated, source of phosphorus enrichment in coastal waters (H2).

**Fig. 3:**
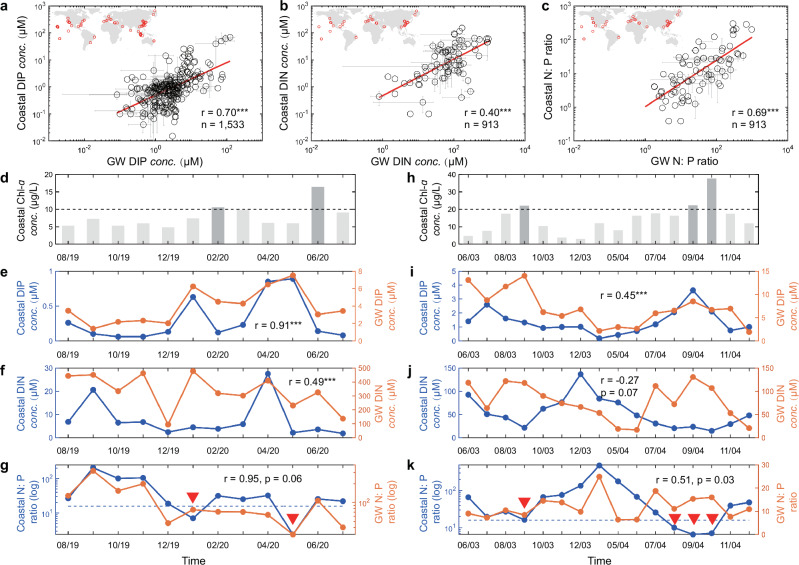
Coastal groundwater is identified as the widespread source of “excess DIP” accelerating recent coastal algal blooms (CABs). **a**–**c** Global-scale log-log correlation plots between in situ observed groundwater (GW) and adjacent coastal surface water for **a** dissolved inorganic phosphorus (DIP) concentrations (µM), **b** dissolved inorganic nitrogen (DIN) concentrations (µM), and **c** N:P ratios. Solid red lines represent linear regressions. Horizontal and vertical error bars denote the statistical variability of the groundwater and coastal datasets, respectively. Inset images show the global distribution of data collection locations (red open circles). **d**–**k** Time-series field observations capturing nutrient dynamics and CABs at two selected representative sites: Tolo Harbour, Hong Kong, China^49^ (**d**–**g**, *n* = 12 sampling campaigns), and Delaware Bay, USA^50,64^ (**h**–**k**, *n* = 16 sampling campaigns). Algal bloom dynamics (**d**, **h**): variations in coastal chlorophyll-*a* (Chl-*a*) concentrations (µg L⁻¹). Dark gray bars denote the local baseline threshold concentrations of CABs (dashed horizontal lines: 10 µg L^−1^ for Hong Kong; 20 µg L^−1^ for Delaware Bay). Nutrient trajectories (**e**, **f**, **i**, **j**): temporal co-variations of DIP concentrations (**e**, **i**) and DIN concentrations (**f**, **j**) in coastal water (blue filled circles and lines, left axes) versus groundwater (orange filled circles and lines, right axes). Stoichiometric shifts (**g**, **k**): temporal tracking of N:P ratios (log-scaled) in coastal water (blue) and groundwater (orange). Dashed blue horizontal lines indicate the Redfield N:P ratio of 16. Red vertical arrows highlight specific months where the coastal water shifted to a phosphorus-surplus state (N:P < 16). The *r* represents Pearson’s correlation coefficient; *p*-values indicate statistical significance (***p < 0.001).

We specifically test hypothesis (H2) through a global synthesis of 1533 paired groundwater and seawater DIP observations from 140 coastal sites (Supplementary Data 1). Groundwater DIP exhibits a strong positive correlation with adjacent seawater DIP (*r* = 0.70, *p* < 0.001, Fig. 3a), indicating that P-rich subterranean inputs overwhelm phytoplankton demand, leaving residual DIP in coastal waters. Conversely, DIN correlations are weaker (*r* = 0.40, *p* < 0.001; Fig. 3b), reflecting pre-discharge nitrogen losses. Consequently, the paired N:P ratio correlates strongly (*r* = 0.69, *p* < 0.001; Fig. 3c), perfectly capturing the dual effect of DIP enrichment and DIN depletion. While we fully acknowledge that total nutrient flux dictates the ultimate ecological impact, utilizing concentration remains a practical approach in this context. Our synthesis of both local (long-term monitoring in Hong Kong) and global scales (31 marginal seas) reveals that groundwater DIP concentrations exhibit vastly greater variance (spanning 2–3 orders of magnitude) than the relatively constrained SGD rates (1–2 orders of magnitude) (Supplementary Fig. 6). Therefore, concentration overall acts as the first-order control that drives the resulting chemical fluxes into coastal ecosystems.

To further validate this mechanism at a high temporal resolution, we turn to long-term records from two geographically and hydrologically distinct sites with recurrent blooms (Fig. 3d–k): Hong Kong, China and Delaware, USA. Both systems receive nutrient-rich groundwater with particularly high DIP relative to DIN^49,50^. Temporal analyses reveal positive correlations between groundwater and adjacent seawater DIP concentrations (*r* = 0.91 for Hong Kong and *r* = 0.45 for Delaware Bay; *p* < 0.001). In stark contrast, correlations for DIN are significantly weaker and variable in sign, confirming that groundwater uniquely drives coastal DIP enrichment without proportionally adding DIN. To translate this chemical coupling into actual ecological impact, we next assess whether these specific pulses of groundwater-derived excess DIP directly trigger CABs. We define bloom events as periods when Chl-*a* concentrations exceed local thresholds values consistent with regional ecological impact criteria (10 µg L^−1^ for Hong Kong^49^. and 20 µg L^−1^ for Delaware Bay^19^ (Fig. 3d, h). Two CABs are thus identified in Hong Kong during February and June 2020 (Fig. 3d) and three events in Delaware during September 2003, August and September 2004 (Fig. 3h). In Hong Kong, sharp increases in groundwater-derived DIP preceding bloom events temporarily alleviated phosphorus limitation. This rapid P-injection enabled phytoplankton to utilize ambient DIN, shifting the system toward nitrogen limitation. Post-bloom observations reveal high residual DIP and depleted DIN levels, matching our conceptual model predictions (Supplementary Fig. 5a) and demonstrate that groundwater-derived DIP directly stimulates blooms. A similar chronological cascade occurs in Delaware Bay, where groundwater DIP enrichment precedes blooms, drives DIN drawdown, and decouples groundwater and coastal nitrogen concentrations, again supporting a phosphorus-triggered bloom mechanism. These site-level observations mirror our global synthesis (Fig. 3a) and provide mechanistic evidence that coastal groundwater can act as a sustained source of excess DIP, systematically lowering N:P ratios and creating conditions that favor CAB initiation and persistence. It is acknowledged that coastal DIP may be exacerbated by global ocean deoxygenation trends^51–55^. Thus, we evaluated whether the excess DIP is also a byproduct of coastal hypoxia cascade. Nevertheless, we found a weaker correlation of DIP and dissolved oxygen (DO) (*r* = –0.37, *p* < 0.001, Supplementary Fig. 3f) than that between groundwater DIP and coastal DIP (Fig. 3a).

Collectively, our results support H2 by identifying coastal groundwater as the origin of the excess DIP driving CABs acceleration. By supplying DIP in excess of phytoplankton stoichiometric demand, such inputs act as a chemical catalyst to unlock bloom potential. This mechanism provides a coherent explanation for the widespread DIP enrichment observed in our global analysis, establishing coastal groundwater as a critical, yet often overlooked, driver of coastal bloom dynamics under contemporary environmental change.

### Spatial and temporal heterogeneity in bloom trends explained by groundwater redox state

Having established coastal groundwater as a major source of excess DIP driving global bloom acceleration, we next address the striking spatial patchwork in CAB trends: why do some regions experience rapid acceleration while others decline or remain stable (Supplementary Fig. 1a)? We hypothesize (H3A) that the regional aquifer redox state acts as a fundamental spatial switch. Specifically, chemically reducing aquifers mobilize and export bioavailable DIP to promote blooms, whereas oxidized aquifers lack this capacity.

**Fig. 4:**
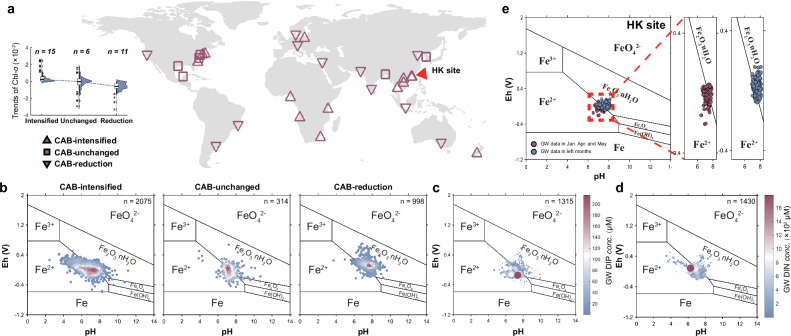
Spatial heterogeneity of coastal algal bloom (CAB) trends explained by groundwater redox states. **a** Global distribution and classification of coastal regions based on long-term trends of chlorophyll-*a* (Chl-*a*): CAB-intensified regions (Chl-*a* trends > 0, *n* = 15), CAB-unchanged regions (Chl-*a* trends $\approx$ 0, *n* = 6), and CAB-reduction regions (Chl-*a* trends < 0, *n* = 11). The inset violin plot shows the distribution of Chl-*a* trend values (×10^−2^) across the three categories. **b** Density scatter plots of groundwater pH and oxidation–reduction potential (ORP, expressed as Eh) superimposed on the iron (Fe) Pourbaix diagram for CAB-intensified (*n* = 2075), CAB-unchanged (*n* = 314), and CAB-reduction (*n* = 998) regions. **c**, **d** Spatial associations between groundwater redox conditions and nutrient concentrations: **c** dissolved inorganic phosphorus (DIP, *n* = 1315) and **d** dissolved inorganic nitrogen (DIN, *n* = 1430). The color gradients indicate the concentrations of groundwater (GW) DIP (µM) and DIN (×10^3^, µM), respectively. **e** Eh–pH conditions of monthly groundwater samples from the Hong Kong (HK) study site plotted on the Fe Pourbaix diagram. Red circles indicate samples collected during January, April, and May 2020; blue circles represent samples collected during the remaining nine months of the year. The inset panels provide magnified views showing the precise distribution of samples relative to the Fe^2+^/Fe_2_O_3_•*n*H_2_O phase boundary.

To test H3A, we classify 32 coastal regions (comprising 3387 observations) based on Chl-*a* trends since 1998: “CAB-intensified” (*n* = 15), “CAB-unchanged” (*n* = 6), and “CAB-reduction” (*n* = 11) (Fig. 4a, Supplementary Data 2). Paired groundwater chemistry analysis reveals a stark contrast. CAB-intensified regions are strongly associated with chemically reducing aquifers (*n* = 2075), characterized by low oxidation–reduction potential (ORP) (Supplementary Fig. 7c) and the presence of dissolved Fe^2+^. Conversely, stable (*n* = 314) or declining (*n* = 998) bloom regions generally exhibit more oxidized or sub-oxidized conditions dominated by Fe^3+^ or transitional phases. Mechanistically, reducing environments promote the reductive dissolution of iron oxides^36^, releasing absorbed DIP. This is evidenced by a strong inverse correlation between ORP and DIP across 1315 groundwater records (Fig. 4c and Supplementary Fig. 7c). While DIN can be present in some reducing environments, removal processes like denitrification often limit its contribution^16^, resulting in lower DIN levels observed in reducing environments (*n* = 1430, Fig. 4d and Supplementary Fig. 7c). Consequently, reducing aquifers distinctly export high DIP, low-N:P groundwater. This large-scale spatial association confirms H3A: the pre-existing aquifer redox state serves as a key long-term vulnerability factor that dictates baseline DIP export, explaining the global spatial heterogeneity of blooms.

However, the spatial vulnerability only provides the potential for DIP release. The actual ecological impact depends on the timing and intensity of DIP fluxes, and whether the receiving coastal water is sensitive at that time. We therefore propose a dual temporal hypothesis (H3B): the stimulatory effect of groundwater DIP is governed by short-term redox fluctuations (which regulate the timing and magnitude of DIP release pulses) and long-term shifts in coastal nutrient limitation regimes (which determine the ecological responsiveness to this input).

To elucidate the short-term dynamics that trigger individual blooms within vulnerable regions, we analyzed high-frequency, year-long monitoring data from the Hong Kong site (Fig. 4e). In the 1–2 months preceding two major algal bloom events (February and June 2020, Fig. 3d), groundwater conditions shifted decisively into the Fe²⁺ stability field in the iron Pourbaix diagram. This short-term fluctuation toward more strongly reducing conditions coincided precisely with a significant pre-bloom spike in groundwater DIP concentrations (Fig. 3d). Thus, within aquifers possessing reduction potential, short-term redox dynamics act as the acute trigger for DIP release pulses and subsequent bloom initiation.

Concurrently, multi-decadal nutrient data validate the long-term dynamic of H3B (Supplementary Fig. 8). Around the year 2000, many coastal systems transitioned from widespread nitrogen limitation to phosphorus limitation. Under P-limited conditions, coastal ecosystems became acutely sensitive to groundwater-derived DIP. The synergy between short-term triggers and long-term nutrient regimes fully explains the catalytic role of groundwater DIP. For instance, during the P-limited period (circa 2000–2019), elevated anthropogenic nutrient loads^56^ combined with short-term DIP pulses from reducing aquifers collectively drove bloom acceleration. Conversely, in recent years, effective nutrient management has reduced nitrogen inputs, shifting systems back toward N-limitation and thereby diminishing the stimulatory effect of groundwater DIP.

Together, these findings validate our integrated mechanism (H3A and H3B): aquifer redox state exerts spatial control over baseline DIP export potential, while the actual realization of blooms is governed by a dual temporal mechanism of short-term redox dynamics (triggering release) and long-term nutrient limitation shifts (defining the sensitive period). This framework comprehensively explains both the global heterogeneity in bloom trends and their changing dynamics over time. The ecological implications are significant: groundwater redox conditions are shaped by both natural processes and human activities, such as land‑use change, groundwater extraction, and climate‑driven shifts in recharge patterns^57–61^. Future alterations to aquifer geochemistry by these factors could inadvertently expand the coastal regions vulnerable to phosphorus‑driven blooms.

In summary, this study resolves the longstanding paradox of accelerating CABs despite decades of nutrient management by identifying coastal groundwater as a critical, yet previously overlooked, geochemical catalyst. Using a global, multi-decadal synthesis, we demonstrate that a sustained rise in DIP, coupled with declining N:P ratios, is the dominant chemical signal driving bloom acceleration (H1). We trace this excess DIP to anoxic coastal groundwater which acts as a disproportionate supplier of low-N:P-ratio nutrients (H2). This mechanism is validated through both global datasets and site-specific time series. Crucially, our integrated framework explains both the spatial and temporal heterogeneity of these blooms: regional aquifer geochemistry acts as a spatial switch, where reducing conditions promote DIP export and bloom vulnerability (H3A); meanwhile, the ecological realization of this DIP flux is temporally gated (H3B), maximizing its catalytic impact during periods of widespread phosphorus limitation and diminishing as systems revert to nitrogen limitation.

These findings carry three pivotal implications for global change and coastal sustainability research. First, they challenge traditional, surface-centric nutrient management^58^. Because groundwater can continuously fuel CABs even where surface nitrogen inputs have been strictly reduced, future monitoring and regulatory frameworks must explicitly integrate subsurface fluxes^29^ to capture the true drivers of bloom dynamics. Second, linking aquifer redox state to nutrient stoichiometry provides a predictive tool for assessing regional vulnerability. As climate change, sea-level rise, altered recharge patterns, and intensified groundwater extraction may cooperatively shift coastal aquifers toward more reducing conditions^62,63^, the mobilization of legacy phosphorus will likely expand the geographic footprint of bloom-prone regions. Tracking aquifer geochemistry will be essential for anticipating ecological tipping points in coastal systems. Third, current coastal ecosystem models heavily prioritize surface inputs, creating a critical blind spot regarding subsurface nutrient stoichiometry^29,58^. Incorporating groundwater-derived DIP and its governing redox mechanisms into next-generation biogeochemical models will drastically improve predictions of: (i) bloom frequency and severity under climate change, (ii) nutrient–oxygen dynamics and hypoxia risk, and (iii) harmful species succession. Ultimately, by unmasking groundwater’s catalytic role in bloom acceleration, our study demands a comprehensive rethinking of dual-nutrient regulation and coastal protection in a climate-driven, increasingly phosphorus-sensitive world.

## Methods

### Data sources

The E.U. Copernicus Marine Environment Monitoring Service (CMEMS) provides a series of global reanalysis products encompassing physical, chemical and biological marine variables at daily, monthly and yearly scales covering decades’ span. The dataset is available at https://data.marine.copernicus.eu/products. We used six physical marine variables, including eastward and northward ocean current velocity, water temperature, water salinity, ocean bathymetry (archived from CMEMS Global Ocean Physical Multiyear Product) and Secchi depth of sea water (archived from Global Ocean Color, Bio-Geo-Chemical, L4 from Satellite Observations), to construct global coastal water stability dataset at monthly scale. We also incorporated wind speed data from the 5th generation European Centre for Medium-Range Weather Forecasts (ECMWF) atmospheric reanalysis of the global climate (ERA5) (https://www.ecmwf.int/en/forecasts/dataset/ecmwf-reanalysis-v5). ERA5 is produced by the Copernicus Climate Change Service (C3S) at ECMWF. We resized all physical data to 1/24° resolution for following calculation and analysis. To explore global CABs and nutrients dynamics, we used monthly chlorophyll mass concentration, nitrate mole concentration, phosphate mole concentration and dissolved oxygen mole concentration from CMEMS Global Ocean Biogeochemistry Multiyear Product (GLOBAL_MULTIYEAR_BGC_001_029) and calculated their decadal trends. This is a model-based hindcast generated by the NEMO3.6-PISCESv2 coupled physical-biogeochemical model. To match with the spatial resolution of constructed global coastal water stability, we also resized the resolution of four chemical and biological marine variables into 1/24° resolution. All data were processed using Matlab.

To compile a global database of paired groundwater-to-coastal DIP and DIN concentrations, we conducted a systematic literature review following a predefined PRISMA flowchart (Supplementary Fig. 9). In brief, an initial literature search was performed using the Web of Science, Scopus, and Google Scholar databases with keywords including (“groundwater” OR ‘aquifer’ or ‘beach groundwater’ Or ‘beach aquifer’ OR “submarine groundwater discharge”) AND (“nutrient” OR “phosphate” OR “DIP” OR “phosphorus”) AND (“coastal” OR “nearshore” OR “seawater”). This search encompassing publications from [Start Year, e.g., 1970] to [End Year, e.g., 2023], yielded over 620 candidate studies. Studies were rigorously screened against the following criteria to ensure data quality and comparability: The seawater sample must have been collected within 2~3 km of the groundwater discharge point or groundwater sampling site to ensure a direct hydrological connection. The groundwater and adjacent seawater samples for each valid station must have been collected within a maximum time window of one month to minimize the influence of temporal variations in discharge or environmental conditions. Studies must report measurable DIP and DIN concentrations for both groundwater and the adjacent coastal water sample. After this screening process, 33 studies met all inclusion criteria and were selected for data extraction. Specially, groundwater of those sites is majorly characterized by saline water. From these 33 studies, we extracted individual paired data points (groundwater DIP concentration and its corresponding coastal seawater DIP concentration). In total, 1533 DIP and 913 DIN paired data points were compiled, forming a global meta-analysis database (Fig. 3A–C, Supplementary Data 1). To support temporal trend analysis, we incorporated high-resolution, in-situ time-series data of groundwater DIP, DIN, coastal DIP, DIN, and chlorophyll-a from field observations in Hong Kong, China^49^ (Fig.3D) and Delaware, USA^50,64^ (Fig.3E). Furthermore, to assess geochemical controls on nutrient speciation, we compiled a global database of 3387 paired groundwater redox potential (Eh) and pH measurements from 32 sites (Fig. 4A, Supplementary Data 2). Subsets of this geochemical database include 1315 and 1430 entries with concurrently measured groundwater DIP and DIN concentrations, respectively (Fig. 4C, D).

### Calculation of global coastal water stability

By deploying process-based mathematical models of coastal water flow and eutrophication^65^, we generated a database of the global coastal water stability. The models depict the ecological response of algal growth to physical environmental constraints. In general, a stable water body is essential for algal blooms. In a flushed tidal inlet with a low flow velocity, i.e., coastal regions, the water column can be simplified to a two-layer (euphotic and aphotic zone) system^66,67^. The production and loss of phytoplankton biomass are respectively determined as the algal growth that only occurs in the euphotic zone and as the mortality and predation, turbulent diffusion, and algal sinking in the aphotic zone^68^. We defined water depth < 30 m as our coastal regions using the ocean bathymetry data from Copernicus Marine Reanalysis data, followed by USEPA^19^. Based on these criteria, a total of 303,212 coastal grid cells were taken into account for the calculation of global coastal water stability.

Normally, vertical turbulent diffusivity (*E*) serves as a measure of vertical mixing in a water column. In non-stratified conditions (*E*_*0*_), vertical mixing can be divided into wind-induced mixing (*E*_*W0*_) at the top layer and tidal mixing (*E*_*T0*_) generated by tidal currents (${E}_{0}={E}_{W0}+{E}_{T0}$). The presence of vertical density stratification acts as a barrier to vertical mixing, thereby negatively impacting *E*_*0*_. Temperature and salinity gradients are the primary drivers of density stratification. When temperature and/or salinity gradients occur, *E* decreases due to damping effects. To quantify the stratified state, a bulk Richardson number (*Ri*) is introduced. *E* thus can be parameterized using *E*_*0*_ and *Ri* with the empirical equation:

1 $$E=\frac{{E}_{0}}{(1+\alpha {Ri})^{\beta }}$$

where empirical constants, *α* and *β*, are 3.33 and 1.5, respectively^69^.

In detail, *E*_*W0*_ can be derived from the 10-m wind speed (ERA5, *W*_*10*_, 4.2 m s^−1^ is set as a breakpoint), and *E*_*T0*_ can be determined by the depth-average maximum velocity (*U*_*d*_) and mean water depth (*H*), so *E*_*0*_ is determined by Eq. 2^68^:

2 $${E}_{0}=\left\{\begin{array}{c}4.3\times {10}^{-4}{W}_{10}^{2}+1.59\times {10}^{-3}{U}_{d}H\,({W}_{10} > 4.2\,m/s)\\ 1.02\times {10}^{-4}{W}_{10}^{3}+1.59\times {10}^{-3}{U}_{d}H\,({W}_{10}\le 4.2\,m/s)\end{array}\right.$$

where we calculated eastward ocean current velocity and northward ocean current velocity and then determined *U*_*d*_ by identifying the maximum velocity from a total of 50 depths.

We used standard method to calculate seawater density^66^ with the water temperature (*T*) and salinity (*S*). For *T* ranging from 0 to 40 °C and *S* ranging from 0.5 to 43 salinity, seawater density (*ρ*) in unit of kg m^−3^ is given by Eq. (3):

3 $$\rho \left(T,S\right)=\,{\rho }_{0}+{AS}+B{S}^{3/2}+C{S}^{2}$$

where:

$$A=	8.24493\times {10}^{-1}-4.0899\times {10}^{-3}T+7.6438\times {10}^{-5}{T}^{2} \\ 	 -8.2467\times {10}^{-7}{T}^{3}+5.3875\times {10}^{-9}{T}^{4},$$

$$B=\,-5.72466\times {10}^{-3}+1.0227\times {10}^{-4}T-1.6546\times {10}^{-6}{T}^{2},$$

$C=4.8314\times {10}^{-4}$, and ρ_0_ is the freshwater density:

$${\rho }_{0}=	999.842594+6.793952\times {10}^{-2}T-9.095290\times {10}^{-3}{T}^{2} \\ 	+1.001685\times {10}^{-4}{T}^{3}-1.120083\times {10}^{-6}{T}^{4}+6.536336\times {10}^{-9}{T}^{5}$$

Thus, we obtained the vertical density difference ($\Delta \rho$) at two given depths, −1.5 m and −6.5 m, thus resulting $\Delta z$ = 5 m. *Ri* is controlled by$\,\Delta \rho$. Also, *Ri* is related to the daily maximum surface velocity (*U*_*s*_) that is influenced by wind (*U*_*sW*_) and tide (*U*_*sT*_). So, *Ri* can be divided into *Ri*_*W*_ and *Ri*_*T*_. Therefore, *Ri* can be expressed as Eq. (4):

4 $${{Ri}}_{X}=\frac{\Delta \rho }{\Delta z}\cdot \frac{g{H}^{2}}{\rho {U}_{{sX}}^{2}}$$

where $\rho$ is determined as 1024 kg m^−3^. *U*_*sW*_ is estimated based on a well-accepted empirical three-percent rule for *W*_*10*_, and *U*_*sT*_ is inferred from the depth-averaged value by assuming the one-seventh power law, 8/7 *U*_*d*_^68^. It should be noted that an upper bound of *Ri* = 15 is set as when *Ri* > 15, the reduction of *E* caused by density stratification can be neglected.

*E* is highly restricted by the critical turbulence threshold (*E*_*c*_) in a water column for an algal bloom likely and *E*_*c*_ can be considerably estimated by the net algal growth rate (*μ*) and the euphotic layer depth (*l*)^21,68^. The *μ* can be obtained by algae growth kinetics and l is related to the light attenuation coefficients to Secchi depth (*Z*_*s*_), given by Eq. (5):

5 $${E}_{c}=\frac{4}{{\pi }^{2}}\cdot \mu {l}^{2}=\frac{4}{{\pi }^{2}}\cdot (0.5{\mu }_{0}{(1.066)}^{T-20{^{\circ }}{{\rm{C}}}}-d)\cdot {(1.9{z}_{s})}^{2}$$

where *μ*_*0*_ is the maximum algal growth rate at 20 °C; *T* is the water temperature in euphotic zone and *d* is the algal decay rate including mortality and flushing. Typically, *d* = 0.1*μ*_*0*_ is often assumed; and *μ*_*0*_ = 2 d^−1^ and *μ*_*0*_ = 0.5 d^−1^ can be estimated for non-mobile species (i.e., diatoms) and mobile species (i.e., dinoflagellates), respectively^67,68^.

When *E/E*_*c*_ < 1, a stable water column significantly favors the algal blooms, otherwise, the strong vertical mixing can highly inhibit algal blooms. In this study, we incorporated both diatoms and dinoflagellates into calculating the *E*_*c*_, which means we identify all the grid pixels with either diatom blooms or dinoflagellate blooms. Following the removal of grids with NaN values from global reanalysis products, we retained a global total of 237,540 coastal grid cells containing valid data for analysis.

### Water stability index

To assess water column stability, we employed two complementary metrics: one quantifying the frequency of stable conditions and the other measuring the continuous intensity of mixing. Theoretically, a stable water column (*E/E*_*c*_ < 1) favors algal bloom development, whereas strong vertical mixing (*E/E*_*c*_ > 1) suppresses it. Accordingly, we first used a binary classification (*E/E*_*c*_ < 1 vs. *E/E*_*c*_ > 1) to evaluate the monthly proportion of the global coastal area under stable conditions. To further incorporate the magnitude of stability or mixing, we also computed the monthly global spatial average of log₁₀(*E/E*_*c*_), generating a continuous annual time series that complements the binary frequency analysis.

### Determination of the percentage of sites under DIP limitation

The percentage of sites under DIP limitation was determined monthly as the proportion of grid cells where the N:P flux ratio exceeded the Redfield ratio of 16:1. Monthly values were averaged annually to quantify their interannual variability.

### Exploring grid-scale covariance trends between Chl-a concentrations and DIP, DIN concentrations and stable water months

To quantify the global prevalence of synergistic trends between Chl-a concentrations and DIP, DIN concentrations and water column stability, we analyzed 237,540 coastal grid cells (0.083° resolution) over five timespans, from 1998 to 2002, from 1998 to 2007, from 1998 to 2012, from 1998 to 2017 and from 1998 to 2022, respectively. For each grid cell and time span, we computed the linear trend (slope) for each variable (Chl-a, DIP, DIN, stable water months). Synergistic trends were defined as a statistically significant (*p* < 0.05), directionally congruent change between Chl-a and a driver variable, including co-increasing trends (slope > 0) or co-decreasing trends (slope < 0). The fraction of sites exhibiting synergistic trends was then calculated as the ratio of grid cells with congruent significant trends (*p* < 0.05) to the total number of cells with valid data in each time window. Statistical significance was assessed using a two-tailed t-test.

### Reporting summary

Further information on research design is available in the Nature Portfolio Reporting Summary linked to this article.

## Supplementary information

Supplementary Information

Description of Additional Supplementary File

Supplementary Data 1

Supplementary Data 2

Reporting Summary

Transparent Peer Review file

## Data Availability

The global reanalysis products used in the study can be archived from the E.U. Copernicus Marine Environment Monitoring Service (CMEMS). The compiled global groundwater database is included in published articles or open sources which have been summarized in Supplementary Data 1&2.

## Code availability

The codes associated with the main figures in this study are available at 10.24433/CO.2162213.v2 (ref. ^70^).
