# Supplementary material for: Coastal groundwater phosphorus drives global acceleration of algal blooms
Source: Nat Commun. 2026 Jul 16;17:6399. doi: 10.1038/s41467-026-75420-y (PMC13377100; doi:10.1038/s41467-026-75420-y)
Supplement: Supplementary file 1 — Supplementary Information [file 41467_2026_75420_MOESM1_ESM.pdf]

Supplementary Information of

Coastal groundwater phosphorus drives global acceleration of algal blooms

K.H. Cheng[1,2,3]*, Joseph H.W. Lee[4], Jiu Jimmy Jiao[2,5]*, Adina Paytan[6], Donald M. Anderson[7], Weijun Cai[8], Holly A. Michael[8], Mathew A. Charette[9], Shuh-Ji Kao[10], Jin Wu[3,11,12]*, Xin Luo[2,13]*

Affiliations:
[1]School of Life Sciences, The Chinese University of Hong Kong; Shatin, Hong Kong, China.
[2]Department of Earth and Planetary Sciences, The University of Hong Kong; Pokfulam, Hong Kong, China.
[3]School of Biological Sciences, The University of Hong Kong; Pokfulam, Hong Kong, China.
[4]Macau Environmental Research Institute, Macau University of Science and Technology; Macau, China.
[5]The University of Hong Kong, Shenzhen Institution of Research and Innovation (SIRI), Shenzhen, China.
[6]Department of Earth and Planetary Sciences, University of California at Santa Cruz; Santa Cruz, CA, USA.
[7]Biology Department, Woods Hole Oceanographic Institution; Woods Hole, MA, USA.
[8]Department of Geological Sciences, University of Delaware; Newark, DE, USA.
[9]Woods Hole Oceanographic Institution; Woods Hole, MA, USA.
[10]State Key Laboratory of Marine Resources Utilization in South China Sea, Hainan University; Haikou, China.
[11]Institute for Climate and Carbon Neutrality, The University of Hong Kong; Pokfulam, Hong Kong, China.
[12]State Key Laboratory of Agrobiotechnology, The Chinese University of Hong Kong; Shatin, Hong Kong, China.
[13]Sea Space Agent Laboratory, Department of Earth and Planetary Sciences, The University of Hong Kong.

***Corresponding authors:** K.H. Cheng (kaihaocheng@cuhk.edu.hk); J.J. Jiao (jjiao@hku.hk); J. Wu (jinwu@hku.hk); X. Luo (xinluo@hku.hk)

This file includes:

Supplementary Figs. 1-9

Supplementary Notes

Supplementary Figures

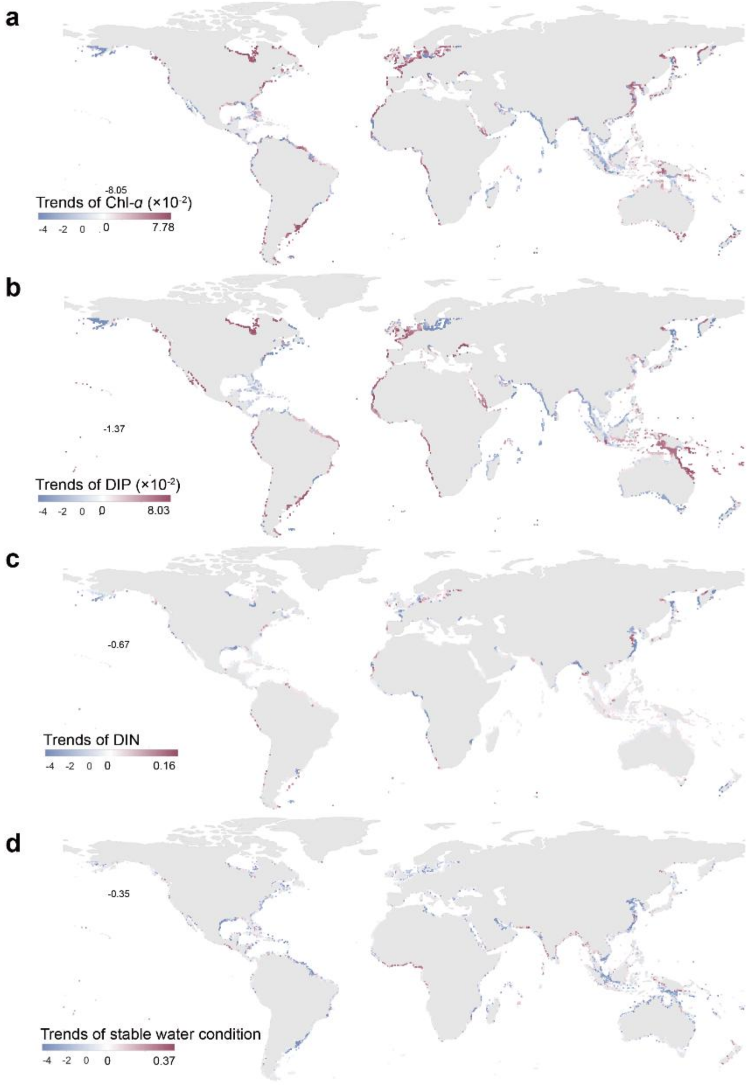

Supplementary Fig. 1. Spatial patterns of trends of chlorophyll-a (Chl-a) concentrations (a), trends of dissolved inorganic phosphorus (DIP) concentrations (b), trends of dissolved inorganic nitrogen (DIN) concentrations (c) and trends of stable water conditions (d) between 1998 and 2022.

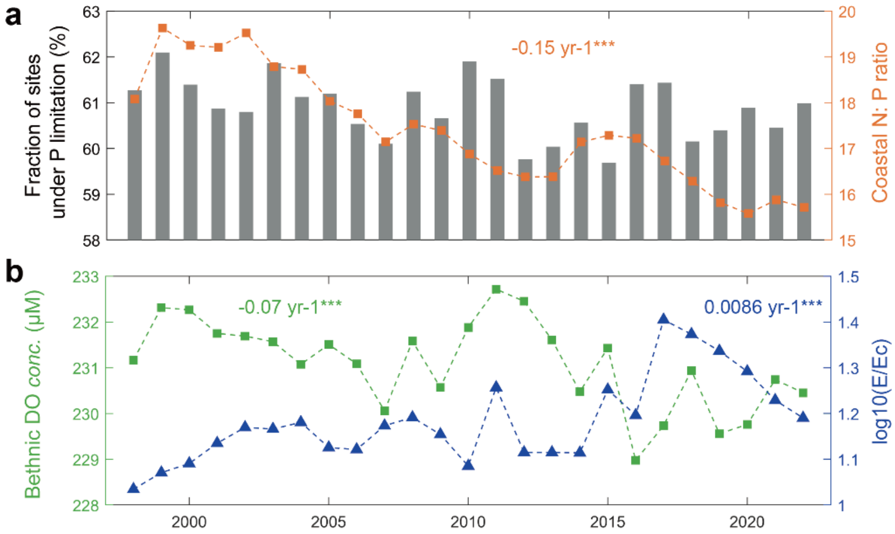

Supplementary Fig. 2. (a) Interannual variability of coastal N: P ratios and the fractions of sites under P limitation (%). (b) Interannual variability of benthic dissolved oxygen (DO) concentrations (μM) and $\log_{10}$(E/Ec). The changing rate per year for N: P ratios, DO and log10(E/Ec) is calculated by the ratio of slope to intercept for the linear regression equation. *** indicates the p value < 0.001.

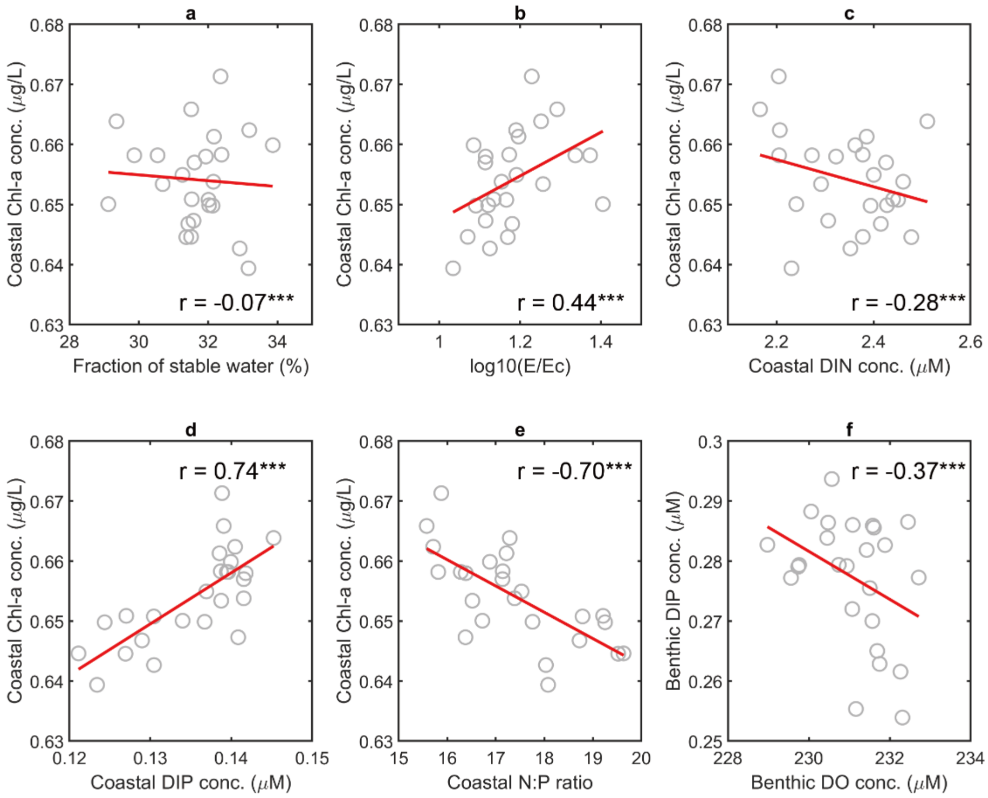

Supplementary Fig. 3. The correlations between annual mean of coastal chlorophyll-a (Chl-a) concentrations (μg L^{-1}) and fraction of stable water (%) (a), log_{10}(E/Ec) (b), coastal dissolved inorganic nitrogen (DIN) concentrations (μM) (c), coastal dissolved inorganic phosphorus (DIP) concentrations (μM) (d), coastal N:P ratios (e) and the correlation between benthic DIP concentrations (μM) and benthic DO concentrations (μM) (f), globally. *** indicates the p value < 0.001.

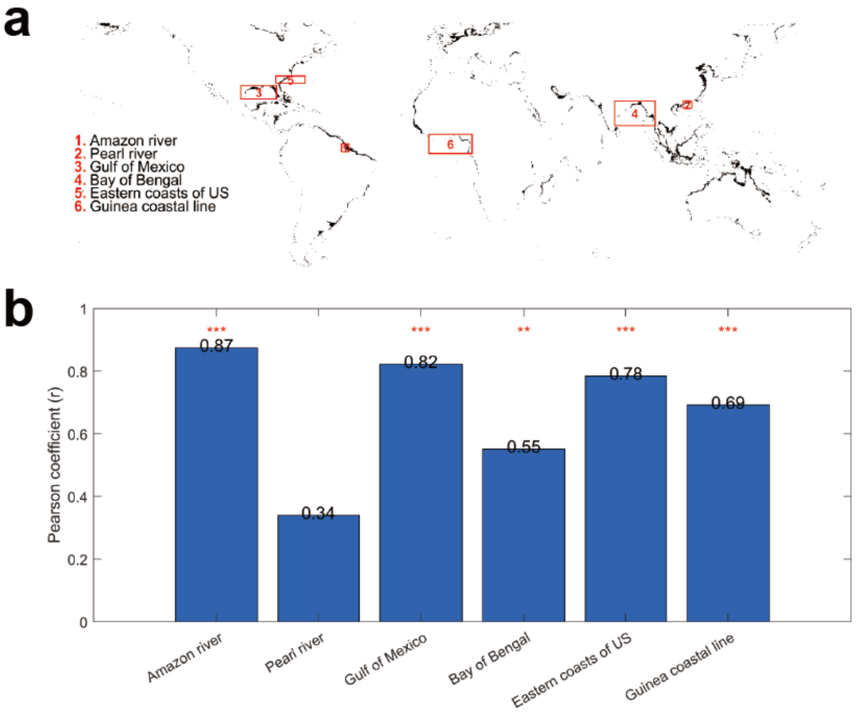

Supplementary Fig. 4. Illustration of the Pearson coefficient (r) between coastal dissolved inorganic phosphorus **(**DIP**)** concentrations and coastal chlorophyll-a **(**Chl-a**)** concentrations based different coastal systems (river-dominated estuaries (Amazon river, Pearl river), enclosed bays (Gulf of Mexico, Bay of Bengal), and open coasts (Eastern coasts of US, Guinea coastal line)).

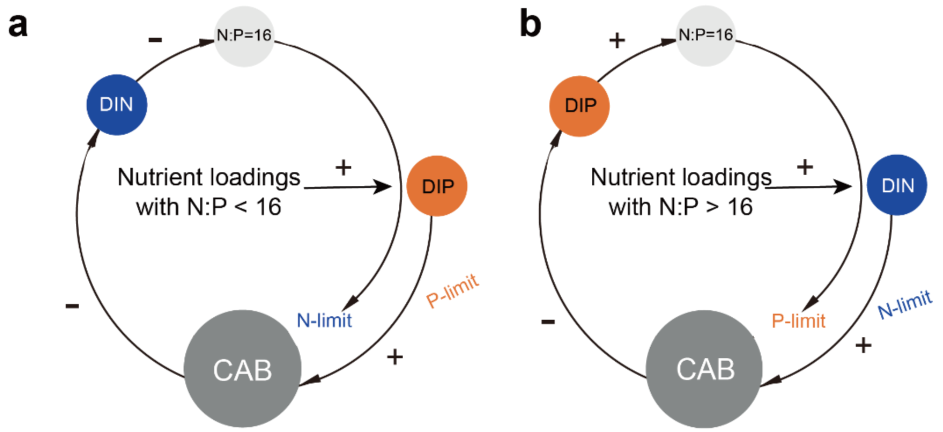

Supplementary Fig. 5. Proposed models to explain the trends of dissolved inorganic phosphorus **(**DIP**),** dissolved inorganic nitrogen **(**DIN**)**, N:P ratios and coastal algal blooms (CABs) with potential nutrient loadings (<16) (a) and potential nutrient loadings (>16) (b).

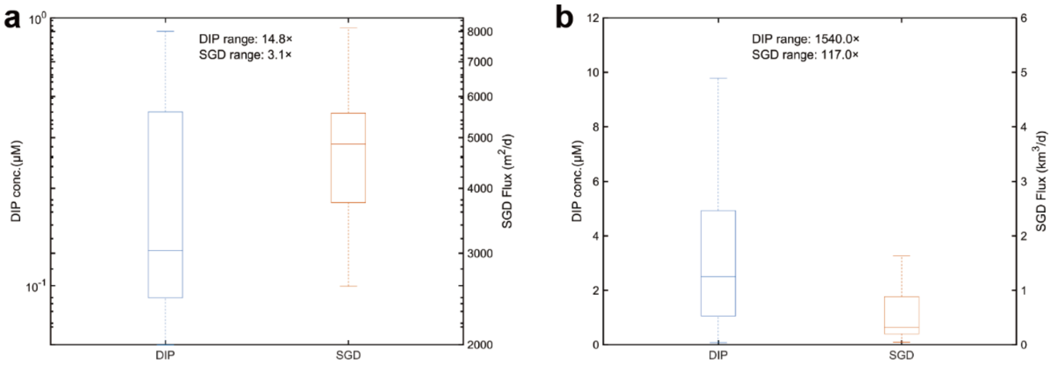

Supplementary Fig. 6. Boxplots of groundwater dissolved inorganic phosphorus (DIP) concentrations and submarine groundwater discharge (SGD) for Hong Kong site (a) and global scale (b). Data for global DIP concentration range is sourced from Fig. 3a, and the estimation of global SGD is given in Supplementary Note.

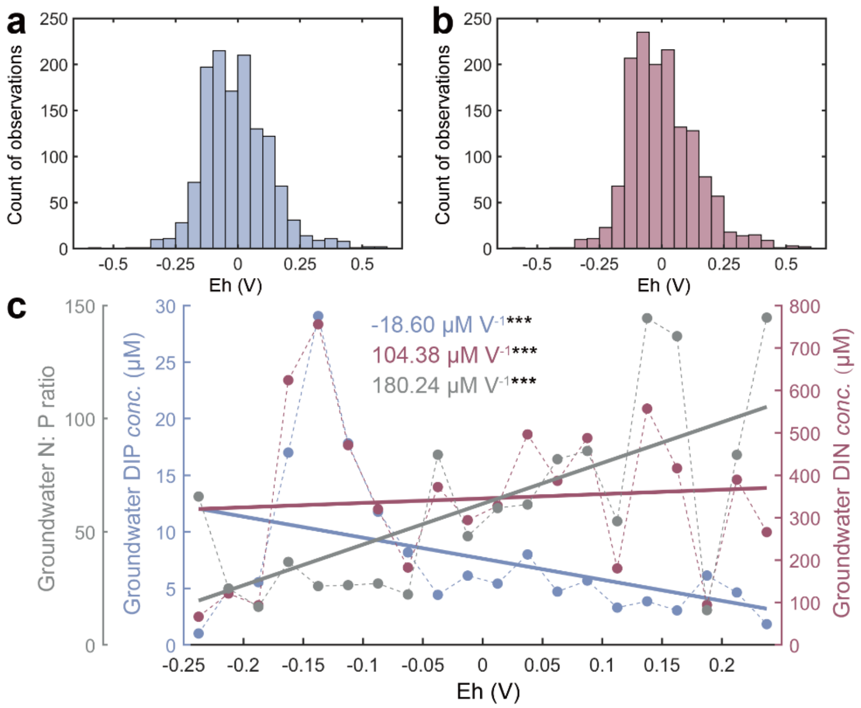

Supplementary Fig. 7. (a) Histogram of field observations of groundwater (GW) oxidation–reduction potential (ORP, Eh (V)) for Fig. 4c. (b) Histogram of field observations of groundwater ORP (Eh (V)) for Fig. 4d. (c) The correlations between groundwater ORP and groundwater dissolved inorganic phosphorus (DIP), dissolved inorganic nitrogen (DIN) concentrations and N:P ratios.

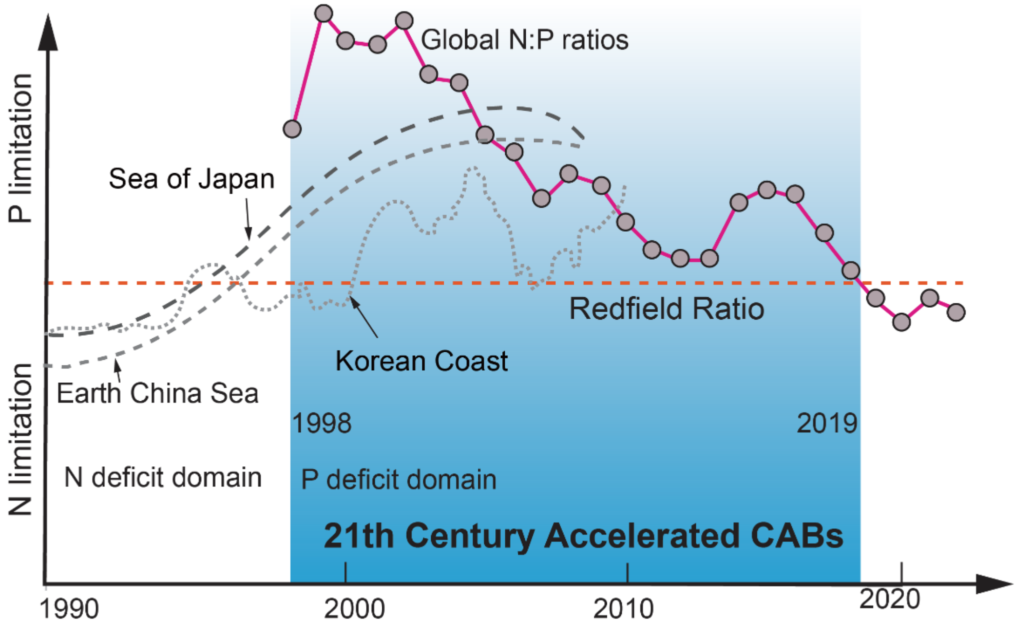

Supplementary Fig. 8. The dynamics of N:P ratios in coastal waters in the past decades in global (this study) and regional scales[1-4], which implies the time-evolving role of groundwater borne dissolved inorganic phosphorus (DIP) in accelerating the coastal algal blooms (CABs) in the new century.

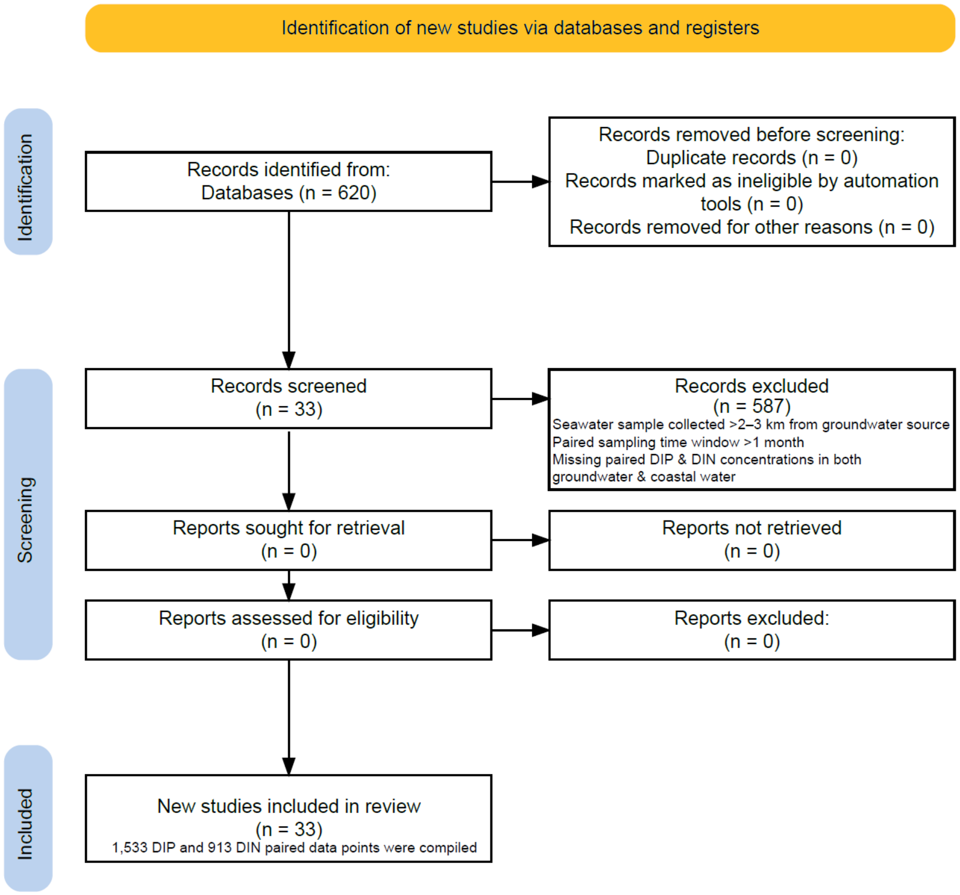

Supplementary Fig. 9. PRISMA flowchart for literature review to compile a global database of paired groundwater-to-coastal dissolved inorganic phosphorus (DIP) and dissolved inorganic nitrogen (DIN) concentrations.

Supplementary Notes

Estimation of Global Submarine Groundwater Discharge (SGD)

To robustly quantify the total submarine groundwater discharge (SGD) across 31 global marginal seas, we conceptualized the total SGD flux as the sum of three primary hydrogeological components: fresh SGD (FSGD), tide-driven recirculated SGD (D_t), and wave-driven recirculated SGD (D_w). The total flux is expressed as:

$$\text{Total SGD} = \text{FSGD} + D_t + D_w \quad (1)$$

1. Fresh Submarine Groundwater Discharge (FSGD)

The global estimates for the terrestrially driven fresh groundwater component (FSGD) were directly extracted from the high-resolution global hydrogeological modeling framework developed by Zhou et al.[71].

2. Tide-Driven Submarine Groundwater Discharge (D_t)

The tide-driven recirculated saline groundwater discharge (D_t) was calculated based on the analytical solutions for coastal aquifer dynamics proposed by Li, et al. [5]. The specific analytical formulation is defined as: where A is the tidal amplitude (m), T_t is the tidal period (s), ω is the tidal frequency (given to be 7.57 x 10^{-5} rad s^{-1}), n_e is the effective porosity of the aquifer (dimensionless), H is the coastal aquifer thickness (m), and S_b represents the beach slope (dimensionless).

$$D_t = \frac{n_e A}{\kappa T_t}\exp(-\alpha)\left[\cos(a)-\sin(a)\right]+\frac{\sqrt{2}n_e}{s_b T_t}\exp(-\sqrt{2}\alpha)\cos(\sqrt{2}\alpha)+\frac{n_e A^2}{s_b T_t} \quad (2)$$

with

$$\kappa = \sqrt{\frac{n_e \omega}{2KH}}$$

$$\alpha = \kappa A / s_b$$

3. Wave-Driven Submarine Groundwater Discharge (D_w)

Similarly, the wave-driven pumping mechanism (D_w), which predominantly forces seawater recirculation through the shallow intertidal zone, was estimated using the empirical analytical framework from Li, et al. [5]:where k is the hydraulic conductivity of the beach sediments (m d^{-1}), S_b is the beach slope, L is the horizontal distance between the wave run-up line and the breaker line (m), σ is the breaking index, H_b is the breaking wave height (m), T_w is the wave period (s), and g is the acceleration due to gravity (9.81 m s^{-2}).

$$D_w = K s_w L = K \times \frac{3\sigma^2 s_b}{8+3\sigma^2} \times \frac{H_b}{\sigma(s_b - \frac{3\sigma^2 s_b}{8+3\sigma^2})} \quad (3)$$

with

$$\sigma = \frac{1.56}{1+\exp(-19.5 s_b)} - 43.8\left[1-\exp(-19 s_b)\right]\frac{H_b}{g T_w^2}$$

4. Global Input Datasets

To apply these analytical solutions globally, we synthesized spatially explicit data for all major unknown variables (hydraulic conductivity, beach slope, wave characteristics, tidal parameters, and aquifer geometries) from state-of-the-art global datasets. The detailed sources are summarized in Supplementary Table S1.

Supplementary Table S1. Summary of global datasets used for SGD calculations.

Parameter	Symbol	Data Source / Description	Reference
Fresh SGD	FSGD	Global high-resolution terrestrial groundwater discharge model.	Zhou, et al. [6]
Beach Permeability	*k*	Global dataset of coastal sediment permeability.	Moosdorf, et al. [7]
Wave Height Period	H_s, T_w	NOAA NCEP WaveWatch III (WW3) Hindcasts (Phase 2).	Tolman [8]
Beach Slope	S_b	Global dataset of nearshore slopes.	Athanasiou, et al. [9]
Hydraulic Conductivity, Porosity	K, n_e	Global Hydrogeology Maps (GLHYMPS v2.0).	Huscroft, et al. [10]
Tidal Amplitude, Period, Frequency	A, T_t, ω	Empirical Ocean Tide model (EOT20).	Hart-Davis, et al. [11]
Aquifer Thickness	*H*	Global gridded dataset of depth to bedrock and aquifer thickness.	Zamrsky, et al. [12]

5. Spatial Harmonization and Regional Aggregation

Given the varying spatial resolutions of the global input datasets (Table S1), all hydrogeological and oceanographic parameters were first harmonized and mapped onto a standardized global coastline grid. The analytical components (D_t) and (D_w) were computed at each discrete coastal grid cell based on the localized parameters.

To estimate the regional-scale SGD, the cell-by-cell fluxes were spatially integrated (accumulated) along the coastal segments bounding the corresponding marine regions. Following this approach, the total SGD fluxes were aggregated for the following 31 major global marginal seas: Andaman (Burma) Sea, Arabian Sea, Argentine Sea, Bay of Bengal, Bay of Biscay, Black Sea, Caribbean Sea, Celebes Sea, Coral Sea, East China Sea, Great Australian Bight, Gulf of Aden, Gulf of California, Gulf of Guinea, Gulf of Mexico, Gulf of Oman, Gulf of Thailand, Japan Sea, Mediterranean Sea, North Brazil Shelf, Peru-Chile Trench, Persian Gulf, Philippine Sea, Red Sea, South Atlantic Bight, South Africa Shelf, South Brazil Shelf, South China Sea, Tasman Sea, Timor Sea, and Yellow Sea. *(Note: The list of marginal seas is arranged alphabetically for clarity).*

References

1 Dai, Y. *et al.* Coastal phytoplankton blooms expand and intensify in the 21st century. *Nature* **615**, 280-284 (2023).

2 Anderson, D. M., Cembella, A. D. & Hallegraeff, G. M. Progress in understanding harmful algal blooms: paradigm shifts and new technologies for research, monitoring, and management. *Annual review of marine science* **4**, 143-176 (2012).

3 Kim, T.-W., Lee, K., Najjar, R. G., Jeong, H.-D. & Jeong, H. J. Increasing N abundance in the northwestern Pacific Ocean due to atmospheric nitrogen deposition. *Science* **334**, 505-509 (2011).

4 Deutsch, C. & Weber, T. Nutrient ratios as a tracer and driver of ocean biogeochemistry. *Annual review of marine science* **4**, 113-141 (2012).

5 Li, L., Barry, D., Stagnitti, F. & Parlange, J. Y. Submarine groundwater discharge and associated chemical input to a coastal sea. *Water Resources Research* **35**, 3253-3259 (1999).

6 Zhou, Y., Sawyer, A. H., David, C. H. & Famiglietti, J. S. Fresh submarine groundwater discharge to the near-global coast. *Geophysical Research Letters* **46**, 5855-5863 (2019).

7 Moosdorf, N., Tschaikowski, J., Kretschmer, D. & Reinecke, R. A global coastal permeability dataset (CoPerm 1.0). *Scientific Data* **11**, 893 (2024). https://doi.org/10.1038/s41597-024-03749-4

8 Tolman, H. L. User manual and system documentation of WAVEWATCH III TM version 3.14. *Technical note, MMAB contribution* **276**, 2009 (2009).

9 Athanasiou, P. *et al.* Global distribution of nearshore slopes with implications for coastal retreat. *Earth Syst. Sci. Data* **11**, 1515-1529 (2019). https://doi.org/10.5194/essd-11-1515-2019

10 Huscroft, J., Gleeson, T., Hartmann, J. & Börker, J. Compiling and mapping global permeability of the unconsolidated and consolidated Earth: GLobal HYdrogeology MaPS 2.0 (GLHYMPS 2.0). *Geophysical Research Letters* **45**, 1897-1904 (2018).

11 Hart-Davis, M. G. *et al.* EOT20: a global ocean tide model from multi-mission satellite altimetry. *Earth Syst. Sci. Data* **13**, 3869-3884 (2021). https://doi.org/10.5194/essd-13-3869-2021

12 Zamrsky, D., Oude Essink, G. H. P. & Bierkens, M. F. P. Estimating the thickness of unconsolidated coastal aquifers along the global coastline. *Earth Syst. Sci. Data* **10**, 1591-1603 (2018). https://doi.org/10.5194/essd-10-1591-2018
