# Supplementary material for: Coastal groundwater phosphorus drives global acceleration of algal blooms
Source: Nat Commun. 2026 Jul 16;17:6399. doi: 10.1038/s41467-026-75420-y (PMC13377100; doi:10.1038/s41467-026-75420-y)
Supplement: Supplementary file 2 — Description of Additional Supplementary File [file 41467_2026_75420_MOESM2_ESM.docx]

**Description of Additional Supplementary Files**

**Supplementary Data 1:** Global database of paired groundwater-tocoastal DIP and DIN concentrations.

**Supplementary Data 2:** Global database of paired groundwater redox potential (Eh) and pH measurements.
