# Supplementary material for: Coastal groundwater phosphorus drives global acceleration of algal blooms
Source: Nat Commun. 2026 Jul 16;17:6399. doi: 10.1038/s41467-026-75420-y (PMC13377100; doi:10.1038/s41467-026-75420-y)
Supplement: Supplementary file 5 — Reporting Summary [file 41467_2026_75420_MOESM5_ESM.pdf]

Corresponding author(s): K.H. CHENG

Last updated by author(s): Jun 30, 2026

Reporting Summary

Nature Portfolio wishes to improve the reproducibility of the work that we publish. This form provides structure for consistency and transparency in reporting. For further information on Nature Portfolio policies, see our Editorial Policies and the Editorial Policy Checklist.

Statistics

For all statistical analyses, confirm that the following items are present in the figure legend, table legend, main text, or Methods section.

n/a	Confirmed	
☒	☐	The exact sample size (*n*) for each experimental group/condition, given as a discrete number and unit of measurement
☒	☐	A statement on whether measurements were taken from distinct samples or whether the same sample was measured repeatedly
☐	☒	The statistical test(s) used AND whether they are one- or two-sided<br>*Only common tests should be described solely by name; describe more complex techniques in the Methods section.*
☒	☐	A description of all covariates tested
☒	☐	A description of any assumptions or corrections, such as tests of normality and adjustment for multiple comparisons
☒	☐	A full description of the statistical parameters including central tendency (e.g. means) or other basic estimates (e.g. regression coefficient) AND variation (e.g. standard deviation) or associated estimates of uncertainty (e.g. confidence intervals)
☒	☐	For null hypothesis testing, the test statistic (e.g. *F*, *t*, *r*) with confidence intervals, effect sizes, degrees of freedom and *P* value noted<br>*Give P values as exact values whenever suitable.*
☒	☐	For Bayesian analysis, information on the choice of priors and Markov chain Monte Carlo settings
☒	☐	For hierarchical and complex designs, identification of the appropriate level for tests and full reporting of outcomes
☒	☐	Estimates of effect sizes (e.g. Cohen's *d*, Pearson's *r*), indicating how they were calculated

Our web collection on statistics for biologists contains articles on many of the points above.

Software and code

Policy information about availability of computer code

Data collection

The E.U. Copernicus Marine Environment Monitoring Service (CMEMS) provides a series of global reanalysis products encompassing physical, chemical and biological marine variables at daily, monthly and yearly scales covering decades' span. The dataset is available at https://data.marine.copernicus.eu/products. We used six physical marine variables, including eastward and northward ocean current velocity, water temperature, water salinity, ocean bathymetry (archived from CMEMS Global Ocean Physical Multiyear Product) and Secchi depth of sea water (archived from Global Ocean Color, Bio-Geo-Chemical, L4 from Satellite Observations), to construct global coastal water stability dataset at monthly scale. We also incorporated wind speed data from the 5th generation European Centre for Medium-Range Weather Forecasts (ECMWF) atmospheric reanalysis of the global climate (ERA5) (https://www.ecmwf.int/en/forecasts/dataset/ecmwf-reanalysis-v5). ERA5 is produced by the Copernicus Climate Change Service (C3S) at ECMWF. We resized all physical data to 1/24°resolution for following calculation and analysis. To explore global CABs and nutrients dynamics, we used monthly chlorophyll mass concentration, nitrate mole concentration, phosphate mole concentration and dissolved oxygen mole concentration from CMEMS Global Ocean Biogeochemistry Multiyear Product (GLOBAL_MULTIYEAR_BGC_001_029) and calculated their decadal trends. This is a model-based hindcast generated by the NEMO3.6-PISCESv2 coupled physical-biogeochemical model. To match with the spatial resolution of constructed global coastal water stability, we also resized the resolution of four chemical and biological marine variables into 1/24°.
To compile a global database of paired groundwater-to-coastal DIP and DIN concentrations, we conducted a systematic literature review following a predefined PRISMA flowchart (Supplementary Fig. 9). In brief, an initial literature search was performed using the Web of Science, Scopus, and Google Scholar databases with keywords including ("groundwater" OR 'aquifer' or 'beach groundwater' Or 'beach aquifer' OR "submarine groundwater discharge") AND ("nutrient" OR "phosphate" OR "DIP" OR "phosphorus") AND ("coastal" OR "nearshore" OR "seawater"). This search encompassing publications from [Start Year, e.g., 1970] to [End Year, e.g., 2023], yielded over 620 candidate studies. Studies were rigorously screened against the following criteria to ensure data quality and comparability: The seawater sample must have been collected within 2~3 km of the groundwater discharge point or groundwater sampling site to ensure a direct hydrological connection. The groundwater and adjacent seawater samples for each valid station must have been collected within a maximum time window of one month to

minimize the influence of temporal variations in discharge or environmental conditions. Studies must report measurable DIP and DIN concentrations for both groundwater and the adjacent coastal water sample. After this screening process, 33 studies met all inclusion criteria and were selected for data extraction. Specially, groundwater of those sites is majorly characterized by saline water. From these 33 studies, we extracted individual paired data points (groundwater DIP concentration and its corresponding coastal seawater DIP concentration). In total, 1,533 DIP and 913 DIN paired data points were compiled, forming a global meta-analysis database (Fig. 3A-C, Supplementary Data 1). To support temporal trend analysis, we incorporated high-resolution, in-situ time-series data of groundwater DIP, DIN, coastal DIP, DIN, and chlorophyll-a from field observations in Hong Kong, China49 (Fig.3D) and Delaware, USA50,65 (Fig.3E). Furthermore, to assess geochemical controls on nutrient speciation, we compiled a global database of 3,387 paired groundwater redox potential (Eh) and pH measurements from 32 sites (Fig. 4A, Supplementary Data 2). Subsets of this geochemical database include 1,315 and 1,430 entries with concurrently measured groundwater DIP and DIN concentrations, respectively (Fig. 4C-D).

Data analysis

All data were processed using Matlab. By deploying process-based mathematical models of coastal water flow and eutrophication66, we generated a database of the global coastal water stability. The models depict the ecological response of algal growth to physical environmental constraints. In general, a stable water body is essential for algal blooms. In a flushed tidal inlet with a low flow velocity, i.e., coastal regions, the water column can be simplified to a two-layer (euphotic and aphotic zone) system67,68. The production and loss of phytoplankton biomass are respectively determined as the algal growth that only occurs in the euphotic zone and as the mortality and predation, turbulent diffusion, and algal sinking in the aphotic zone69. We defined water depth < 30 m as our coastal regions using the ocean bathymetry data from Copernicus Marine Reanalysis data, followed by USEPA19. Based on these criteria, a total of 303,212 coastal grid cells were taken into account for the calculation of global coastal water stability.

For manuscripts utilizing custom algorithms or software that are central to the research but not yet described in published literature, software must be made available to editors and reviewers. We strongly encourage code deposition in a community repository (e.g. GitHub). See the Nature Portfolio guidelines for submitting code & software for further information.

Data

Policy information about availability of data

All manuscripts must include a data availability statement. This statement should provide the following information, where applicable:

- Accession codes, unique identifiers, or web links for publicly available datasets
- A description of any restrictions on data availability
- For clinical datasets or third party data, please ensure that the statement adheres to our policy

The global reanalysis products used in the study can be archived from the E.U. Copernicus Marine Environment Monitoring Service (CMEMS). The compiled global groundwater database is included in published articles or open sources which have been summarized in Supplementary Data 1&2.

Research involving human participants, their data, or biological material

Policy information about studies with human participants or human data. See also policy information about sex, gender (identity/presentation), and sexual orientation and race, ethnicity and racism.

Reporting on sex and gender: Not applicable.

Reporting on race, ethnicity, or other socially relevant groupings: Not applicable.

Population characteristics: Not applicable.

Recruitment: Not applicable.

Ethics oversight: Not applicable.

Note that full information on the approval of the study protocol must also be provided in the manuscript.

Field-specific reporting

Please select the one below that is the best fit for your research. If you are not sure, read the appropriate sections before making your selection.

☐ Life sciences ☐ Behavioural & social sciences ☒ Ecological, evolutionary & environmental sciences

For a reference copy of the document with all sections, see nature.com/documents/nr-reporting-summary-flat.pdf

Ecological, evolutionary & environmental sciences study design

All studies must disclose on these points even when the disclosure is negative.

Study description

This global, multi-decadal study resolves a long-standing ecological paradox: why coastal algal blooms (CABs) continue to accelerate worldwide even in regions where nitrogen pollution has been strictly regulated. The research identifies anoxic coastal groundwater as a critical, previously overlooked geochemical catalyst delivering excess phosphorus to coastal waters, effectively rewriting our

understanding of the modern coastal nutrient cycle.

Research sample	This study integrates a multi-scale, global dataset combining satellite-derived records, biogeochemical model reanalyses, a literature-compiled global groundwater meta-analysis database, and high-resolution local time-series validation. The research sample is divided into four primary components. Global Spatial-Temporal Analysis: Evaluated physical and chemical parameters across 237,540 coastal sites over a 25-year period (1998–2022). Global Meta-Analysis: Synthesized 1,533 paired groundwater and seawater nutrient observations across 140 coastal sites, alongside a geochemical database of 3,387 regional aquifer measurements. High-Resolution Validation: Analyzed long-term, site-specific time series from two hydrologically distinct, bloom-prone regions: Tolo Harbour (Hong Kong, China) and Delaware Bay (USA).
Sampling strategy	Not applicable.
Data collection	Global reanalysis products were downloaded from the E.U. Copernicus Marine Environment Monitoring Service (CMEMS).
Timing and spatial scale	We used the monthly data covering 1998 to 2022.
Data exclusions	No data were excluded from analysis.
Reproducibility	This study is a meta analysis study. There is no generated field data. All data used were downloaded or sourced from literature review.
Randomization	Not applicable.
Blinding	Not applicable.

Did the study involve field work? ☐ Yes ☒ No

Reporting for specific materials, systems and methods

We require information from authors about some types of materials, experimental systems and methods used in many studies. Here, indicate whether each material, system or method listed is relevant to your study. If you are not sure if a list item applies to your research, read the appropriate section before selecting a response.

Materials & experimental systems

n/a	Involved in the study	
☒	☐	Antibodies
☒	☐	Eukaryotic cell lines
☒	☐	Palaeontology and archaeology
☒	☐	Animals and other organisms
☒	☐	Clinical data
☒	☐	Dual use research of concern
☒	☐	Plants

Methods

n/a	Involved in the study	
☒	☐	ChIP-seq
☒	☐	Flow cytometry
☒	☐	MRI-based neuroimaging

Plants

Seed stocks	Not applicable.
Novel plant genotypes	Not applicable.
Authentication	Not applicable.
