# Supplementary material for: Coastal groundwater phosphorus drives global acceleration of algal blooms
Source: Nat Commun. 2026 Jul 16;17:6399. doi: 10.1038/s41467-026-75420-y (PMC13377100; doi:10.1038/s41467-026-75420-y)
Supplement: Supplementary file 6 — Transparent Peer Review file [file 41467_2026_75420_MOESM6_ESM.pdf]

nature portfolio

Peer Review File

Coastal groundwater phosphorus drives global acceleration of algal blooms

Corresponding Author: Dr K.H. Cheng

This file contains all reviewer reports in order by version, followed by all author rebuttals in order by version.

Version 0:

Reviewer comments:

Reviewer #1

(Remarks to the Author)
This is an interesting and ambitious analysis that aims at demonstrating the importance of groundwater DIP in controlling CABs in shallow (< 30 m) coastal waters globally. It seems unnecessarily complicated by throwing in a side-story about changes in P rock mining and P fertilizer at national scales, which is not well integrated into the rest of the study. The authors pose questions as a series of hypotheses, the first of which is that climate change may be increasing water column stability which in turn may favor phytoplankton accumulation. An alternative hypothesis (H2) is that changes nutrient stoichiometry (DIP vs DIN) may be accelerating CABs. Hypotheses 3 and 4, the most novel, are that stoichiometric changes in these waters are driven by groundwater DIP, and that the spatial variation in this factor is controlled by redox conditions (reducing conditions favor more groundwater DIP). A problem with this framework is that the authors are simultaneously trying to falsify hypotheses and to claim that the conclusions apply on a global scale. A secondary issue is the potential mismatch between scale of available data and the mechanisms being described.

If I understand correctly, the data used in the analyses are either at 1/24 degree resolution or 1/12 degree resolution (for chl-A and nutrients). At the equator, a grid cell of 1/24 degree has an area of around 21.5 km2. At 30 degrees north, it is around 18.6 km2. The areas studied are coastal waters < 30 m deep. For H1, the stability measures employed rely on temperature, salinity and wind values which represent the areas of the gridcells. The stability index, E, is scaled to a threshold value Ec, related to hypothetical growth rates of phytoplankton. It is hard to understand how the stability dynamics of water movement can be adequately represented by this lateral spatial resolution in such shallow systems. Can the authors justify this? Clearly there are significant sub-grid scale dynamics not captured here. Further, the statistical measure employed simply captures whether the proportion of instances that E is either greater or less than EC, ignoring the magnitude of E/Ec. Was any consideration given to alternative measures?

H2 is considerably less problematic, as it amounts to testing whether changing nutrient and Chl-a concentrations in the same grid cells are correlated. Unsurprisingly, Chl-a concentrations are positively correlated with DIP concentrations, both of which are increasing, and negatively correlated with N:P ratios; DIN concentrations are declining overall, and also negatively correlated with Chl-a. The authors make a compelling argument, based on a large review of the groundwater nutrient/redox literature that DIP is becoming more available in coastal groundwaters due to the increased prevalence of reducing conditions, leading to desorption of sediment-bound P. These same conditions support increased denitrification, leading to declining N concentrations, and even lower N:P in groundwater. I found it a little odd that they didn't seem concerned with testing a hypothesis (H3b?) that increasing hypoxia/anoxia in shallow coastal waters may also be releasing P from sediments. They tend to dismiss such sources as "localized and insufficient to explain the coherent DIP signal (34)", yet the cited reference (Wallman, 2010) notes that "The expansion of oxygen minimum zones [Stramma et al., 2008] may increase the benthic fluxes of phosphate at the continental margins. The further expansion of oxygen minimum zones predicted by ocean modeling [Oschlies et al., 2008] may be amplified by the enhanced benthic phosphate release from margin sediments in a positive feedback loop." It seems that this mechanism, enhanced release of sediment bound P in shallow coastal waters due to increased hypoxia/anoxia, is also likely to be relevant, and worth investigating using the same approach taken by the authors.

Figs:
Fig 2: It is worth noting that most of the increase in DIP shown in figure 2 occurs prior to 2010 and then the trend appears to flatten or even decline. Also, figure 2d is not helpful, and somewhat confusing.
Fig 3d: Rather dense. Is it necessary to combine bar charts and xy plots? I don't see an advantage, so would separate them or plot everything as xy plots. In any case, please explain the subtleties in more detail (arrows, shading, etc)
S4: A companion to figure 2d and equally uninformative. These alternative "models" should be explained in a forthright manner in the text. I find them meaningless on their own. If they are included, they should appear as panels a and b to facilitate comparison.
Fig S5a,b: These appear to be frequency histograms of Eh(v), but the text refers to them as histograms of groundwater DIP and DIN "verse groundwater ORP (Eh (V))". I don't understand what this means. Please clarify.
Fig S7: Far too much information being displayed on the same figures. Please break them into separate panels and explain them in associated text.

Other comments:

Line 113: "rules out"? Given the resolution of the available data, perhaps "fails to support" is a better choice of words.
Lines318-330: It is entirely unclear how this section on global fertilizer use and phosphate rock production relates to the main focus of the study. The authors should be aware of much more thorough studies of global P sources and their environmental consequences. See Pei et al. (2016) for a recent example; there are many others. Such analyses might provide more robust means of estimating P to groundwater and coastal waters.
Line 347: Shouldn't it read "E decreases due to damping effects"?
Lines 350-351: Can the authors point out where there is a reference to E and its empirical constants in (53)?
Line 390: diatoms blooms or dinoflagellates blooms -> diatom blooms or dinoflagellate blooms
Throughout: The paper has a few misspellings (e.g. "tones" for "tons"), minor grammatical errors, etc throughout the text and supplemental material.

References
Oschlies, A., K. G. Schulz, U. Riebesell, and Schmittner (2008), Simulated 21st century's increase in oceanic suboxia by CO2-enhanced biotic carbon export, Global Biogeochem. Cycles, 22, GB4008, doi:10.1029/2007GB003147.
Pei, W., Zhao, H., Han, J., Sonne, C., Whelan, M., Lam, S.S., Guo, H., Fan, Z. and Li, M., 2026. Global Anthropogenic Phosphorus Inputs and Future Sustainability Solutions. Environmental Science & Technology.
Stramma, L., G. Johnson, Sprintall, and V. Morholz (2008), Expanding oxygen-minimum zones in the tropical oceans, Science, 320, 655–658, doi:10.1126/science.1153847.
Wallmann, K., 2010. Phosphorus imbalance in the global ocean?. Global Biogeochemical Cycles, 24(4).

(Remarks on code availability)

Reviewer #2

(Remarks to the Author)
This manuscript presents a potentially important and highly original framework linking coastal groundwater phosphorus inputs to the recent intensification of coastal algal blooms (CABs). The study addresses a major scientific question and integrates global nutrient datasets, satellite-derived Chl-a, groundwater geochemistry, and local time-series observations into a coherent hypothesis-driven narrative. In particular, the proposed linkage between coastal aquifer redox conditions, dissolved inorganic phosphorus (DIP) mobilization, and altered coastal nutrient stoichiometry is conceptually novel and could have broad implications for coastal biogeochemistry and eutrophication management.

However, I have several major concerns regarding the robustness of the central conclusions.

First, the manuscript relies heavily on satellite-derived Chl-a trends in optically complex coastal waters (<30 m depth), where Chl-a retrieval uncertainties are often substantial due to suspended sediments, CDOM, shallow-water effects, etc. The authors interpret increasing Chl-a as evidence for "global CAB acceleration" but it remains unclear whether the reported trends reflect true phytoplankton biomass increases or, at least partly, retrieval artifacts associated with changing coastal optical properties.

Second, the manuscript may overstate the causal role of groundwater-derived DIP. While the presented correlations strongly suggest that groundwater can act as an important phosphorus source under reducing conditions, the current analyses do not sufficiently constrain the relative contribution of groundwater phosphorus fluxes compared with other major phosphorus sources such as sediment regeneration, rivers, wastewater, and atmospheric deposition. In particular, sedimentary phosphorus recycling under reducing conditions may produce patterns similar to those attributed here to groundwater inputs. Therefore, the conclusion that groundwater is the dominant driver of recent global CAB acceleration appears stronger than what the available evidence can fully support.

Finally, I encourage the authors to moderate some of the terminology throughout the manuscript. The study primarily analyzes long-term mean Chl-a trends rather than direct bloom metrics (e.g., bloom frequency, duration, or HAB occurrence records). Thus, terms such as "coastal phytoplankton biomass increase" or "enhanced eutrophic conditions" may be more appropriate than repeatedly referring to "global CAB acceleration." Overall, this is an ambitious and potentially high-impact study, but the manuscript would benefit from a more cautious interpretation of the remote sensing evidence and a clearer

assessment of uncertainties and source attribution.

(Remarks on code availability)

Reviewer #3

(Remarks to the Author)
This manuscript addresses an important scientific question concerning the continued acceleration of coastal algal blooms under changing nutrient regimes. The authors propose that DIP derived from coastal groundwater represents a previously underappreciated driver of global coastal algal bloom acceleration. The study integrates multiple lines of evidence, including global reanalysis products, a large synthesis of paired groundwater-seawater nutrient observations, site-specific time series, and groundwater redox data. Overall, the manuscript is well structured, and the sequence of hypotheses is logically developed. The proposed mechanism has important implications for coastal nutrient management. Nevertheless, several aspects of the evidence chain and methodology require further clarification and strengthening before the central conclusion can be fully supported.
Major comments
1. The groundwater evidence should be linked more explicitly to nutrient fluxes rather than concentrations alone. The global groundwater synthesis is based mainly on paired groundwater and adjacent seawater DIP/DIN concentrations. This is valuable, but algal bloom responses depend on nutrient loads or fluxes, not concentrations alone. Groundwater-derived DIP flux depends on both DIP concentration and submarine groundwater discharge rate. Therefore, high groundwater DIP concentration does not necessarily indicate a large contribution to coastal nutrient budgets. Site-level SGD rates or published nutrient flux estimates should be incorporated to demonstrate whether groundwater DIP inputs are quantitatively sufficient to support the observed bloom responses.
2. Although DIP–Chl-a co-occurrence is higher than that of DIN or water-column stability, the substantial co-occurrence values for DIN and stability suggest that these factors may still be important controls in many coastal systems. The manuscript would be strengthened by further characterizing the sites where DIN–Chl-a or stability–Chl-a co-occurrence is strong. For example, the authors could examine whether these sites differ in geographic distribution, nutrient limitation regime, hydrodynamic setting, human nutrient inputs, or groundwater redox conditions. This would help clarify where groundwater-derived DIP is likely to be the dominant control and where other mechanisms may be more important.
3. The two site-specific case studies require clearer justification and more direct presentation of site-level N:P evidence. The Hong Kong and Delaware examples provide useful support because groundwater DIP enrichment appears to precede bloom events and is followed by DIN drawdown. However, the manuscript refers to them as “two contrasting sites” without clearly explaining the basis of the contrast. The authors should specify in what sense these two sites are contrasting, such as climate, geomorphology, hydrodynamics, aquifer type, nutrient sources, or bloom characteristics. In addition, the site-specific groundwater N:P ratios should be reported more clearly to show whether low-N:P groundwater input is directly observed before bloom events. In Fig. 3D, the axis scaling or plotted values of groundwater N:P ratio appear unclear and may be inconsistent with the interpretation of low-N:P groundwater input. The authors should check the calculation, axis labeling, and interpretation of groundwater N:P ratios in this figure.
4. The linkage between groundwater redox state and bloom trends is promising, but the temporal evidence for changes in groundwater redox conditions remains unclear. The manuscript provides a temporal analysis showing that the effect of groundwater-derived DIP may depend on shifts in coastal nutrient limitation regimes. However, the evidence linking aquifer redox state to CAB trends appears to be based mainly on spatial association among regions with different bloom trends. It remains unclear whether groundwater redox conditions themselves have changed over time in regions where blooms have intensified. The authors should clarify whether reducing aquifers represent a pre-existing vulnerability that enhances bloom responses under phosphorus limitation, or whether temporal changes in groundwater redox conditions have directly contributed to increasingly severe blooms.
5. The authors resampled the global datasets to 1/24° resolution for consistency among physical, chemical, and biological variables, but the manuscript does not justify whether this spatial resolution is sufficient to resolve nearshore groundwater discharge signals. Given that SGD and associated nutrient gradients often occur at much smaller spatial scales, the authors should discuss the potential scale mismatch and test whether the results are robust to alternative coastal-zone definitions or spatial resolutions.
Minor comments
1. The sentence “As coastal populations expand and pressures from resource use intensify…, the urgency to understand and mitigate CABs…” is grammatically incomplete because it lacks a main verb. It could be revised as: “As coastal populations expand and pressures from resource use intensify, it is increasingly urgent to understand and mitigate CABs, not only to safeguard ecosystem resilience but also to protect livelihoods in a changing climate.”
2. The phrase “two contrasting sites” should be clarified or replaced with a more specific description, such as “two geographically and hydrologically distinct sites,” if the contrast is explicitly supported.
3. The bloom thresholds used for Hong Kong and Delaware differ. The authors should briefly justify these thresholds and indicate whether the results are sensitive to threshold choice.
4. It’s better to provide more details on the selected 32 coastal regions used for the groundwater redox analysis, preferably in a supplementary table, including location, sample size, redox category, bloom trend category, and data source.
5. It should be clarified whether seasonal variability was considered in the groundwater–seawater nutrient comparison. Groundwater nutrient concentrations and coastal blooms can both vary strongly by season.
6. The authors should more clearly distinguish between groundwater nutrient concentration and groundwater nutrient flux throughout the manuscript.
7. Figure S5 does include groundwater N:P information, but the main text should refer to it more explicitly when discussing low-N:P groundwater inputs.

8. It is not clear whether river-dominated estuaries, enclosed bays, lagoons, and open coasts were analyzed together or separately. These systems have very different hydrodynamic and nutrient cycling characteristics, which may influence the DIP–Chl-a relationship.
9. The authors should also clarify whether the groundwater-seawater paired nutrient database includes both fresh groundwater discharge and recirculated seawater through subterranean estuaries. These two pathways may have different nutrient signatures and management implications.

(Remarks on code availability)

Version 1:

Reviewer comments:

Reviewer #1

(Remarks to the Author)
The authors have adequately addressed the concerns I raised about the previous version of the paper, and I believe it to be a solid contribution to the literature.

(Remarks on code availability)

Reviewer #2

(Remarks to the Author)
The authors have addressed my comments well, I don't have any further questions.

(Remarks on code availability)

Reviewer #3

(Remarks to the Author)
My comments have been well addressed.

(Remarks on code availability)

Open Access This Peer Review File is licensed under a Creative Commons Attribution 4.0 International License, which permits use, sharing, adaptation, distribution and reproduction in any medium or format, as long as you give appropriate

credit to the original author(s) and the source, provide a link to the Creative Commons license, and indicate if changes were made.
In cases where reviewers are anonymous, credit should be given to 'Anonymous Referee' and the source.
The images or other third party material in this Peer Review File are included in the article's Creative Commons license, unless indicated otherwise in a credit line to the material. If material is not included in the article's Creative Commons license and your intended use is not permitted by statutory regulation or exceeds the permitted use, you will need to obtain permission directly from the copyright holder.
To view a copy of this license, visit https://creativecommons.org/licenses/by/4.0/

REVIEWER COMMENTS

Reviewer #1 (Remarks to the Author):

Major comment 1: This is an interesting and ambitious analysis that aims at demonstrating the importance of groundwater DIP in controlling CABs in shallow (< 30 m) coastal waters globally. It seems unnecessarily complicated by throwing in a side-story about changes in P rock mining and P fertilizer at national scales, which is not well integrated into the rest of the study. The authors pose questions as a series of hypotheses, the first of which is that climate change may be increasing water column stability which in turn may favor phytoplankton accumulation. An alternative hypothesis (H2) is that changes nutrient stoichiometry (DIP vs DIN) may be accelerating CABs. Hypotheses 3 and 4, the most novel, are that stoichiometric changes in these waters are driven by groundwater DIP, and that the spatial variation in this factor is controlled by redox conditions (reducing conditions favor more groundwater DIP). A problem with this framework is that the authors are simultaneously trying to falsify hypotheses and to claim that the conclusions apply on a global scale.

Response: We thank the reviewer for the encouragement and insightful comments. We agree that the P rock mining and fertilizer analysis detracted from our core focus. As suggested, we have streamlined the narrative by removing these statements from the main text, Methods, and SI, including the deletion of former Fig. S7. We fully agree with the reviewer's insightful point that global-scale environmental complexities make rigid falsification logically problematic. We acknowledge that our previous tone was overly absolute; our intention was never to dismiss established drivers, which remain critical in many regional contexts. To address this, we have extensively reorganized the main text and eliminated the binary "either/or" rhetoric. First, we simplified the framework by merging the original H1 and H2 into a new H1 (physical vs. chemical drivers), while reassigning the groundwater source to H2, and the spatial/temporal mechanisms to H3A/H3B. Conceptually, we no longer attempt to "falsify" drivers. Instead, we reframe H1 (e.g., climate/physical factors) as the "physical engine" providing favorable environmental windows, and H2 (groundwater DIP) as the indispensable "biogeochemical fuel." By evaluating H1 globally, we highlight the unexplained variance in CAB outbreaks, thereby statistically and ecologically justifying the introduction of H2 and H3 (SGD and redox conditions) as crucial missing drivers. We believe this reframed approach respects the established importance of physical/chemical factors while robustly elevating the novel significance of groundwater DIP.

Major comment 2: A secondary issue is the potential mismatch between scale of available data and the mechanisms being described. If I understand correctly, the data used in the analyses are either at 1/24 degree resolution or 1/12 degree resolution (for chl-A and nutrients). At the equator, a grid cell of 1/24 degree has an area of around 21.5 km2. At 30 degrees north, it is around 18.6 km2. The areas studied are coastal waters < 30 m deep. For H1, the stability measures employed rely on temperature, salinity and wind values which represent the areas of the grid cells. The stability index, E, is scaled to a threshold value Ec, related to hypothetical growth rates of phytoplankton. It is hard to understand how the stability dynamics of water movement can be adequately represented by this

lateral spatial resolution in such shallow systems. Can the authors justify this? Clearly there are significant sub-grid scale dynamics not captured here.

Response: We thank the reviewer for this insightful comment regarding the representation of physical processes at our chosen data resolution. We acknowledge that sub-grid scale dynamics exist in shallow coastal systems. Our use of the ~4 km (1/24°) grid is a practical and necessary choice for a global-scale analysis, justified as follows: The 1/24° resolution represents a common and practical scale for global coastal analyses using reanalysis products. This resolution provides a consistent framework to compute long-term, spatially coherent trends across decades globally, which is the primary objective of our H1 analysis. Finer-resolution global data products with the requisite multi-decadal coverage and consistent physics are not available for the long period (1998-2022) and global extent of our study. The water-column stability metric (E) estimates the turbulent kinetic energy available for mixing, primarily driven by wind stress and buoyancy flux. In many coastal regions, the first-order, synoptic-scale meteorological forcing (wind stress, heat flux) that controls the mixed layer depth and stratification over weekly to monthly timescales relevant to bloom development is adequately captured at this ~4 km scale. While sub-grid bathymetric variations and transient small-scale fronts are not resolved, our analysis targets the large-scale, climate-driven changes in the background physical window favorable or unfavorable for bloom accumulation. The goal of testing H1 is not to simulate local mixing dynamics perfectly but to evaluate whether globally coherent trends in physical stability can explain the observed globally coherent trend in algal biomass. The ~4 km grid allows us to average over some sub-grid noise and identify these large-scale patterns.

Major comment 3: Further, the statistical measure employed simply captures whether the proportion of instances that E is either greater or less than EC, ignoring the magnitude of E/Ec. Was any consideration given to alternative measures?

Response: We thank the reviewer for this pertinent question regarding our stability metric. The binary classification (E>Ec or E<Ec) is indeed a simplification, chosen to translate the continuous physical energy into a clear, ecologically relevant state of a favorable (stratified, E<Ec) or unfavorable (mixed, E>Ec) physical window for phytoplankton accumulation. This approach directly addresses whether the physical conditions were conducive to bloom formation at a given time, which can address our initial H1.

We agree that the continuous magnitude of E/Ec contains additional information. In direct response to the reviewer's suggestion, we have performed a sensitivity analysis using the continuous value of $\log_{10}(E/Ec)$ as an alternative stability index, which directly incorporates the magnitude information. This analysis yielded insightful results that strengthen our interpretation. We recalculated the monthly global spatial average of $\log_{10}(E/Ec)$ for each time step, and then created a complementary continuous yearly-based time series to our original binary proportion ($E/Ec < 1$). In principle, the binary metric tracks the frequency of stable conditions, while the continuous metric is sensitive to the intensity of mixing events. Our results robustly confirm and extend our original findings. The two independent metrics revealed convergent long-term trends. The original binary

proportion of E/Ec < 1 shows a significant decreasing trend (Fig. 2B), indicating that the spatial extent of water columns satisfying the stable condition (E/Ec < 1) has declined globally. The long-term trend of the spatially averaged $\log_{10}$(E/Ec) is positive ($\log_{10}$(E/Ec) > 0 indicates a mixed state on average) and increasing (Supplementary Fig. 2B), indicating a strengthening of the average mixing intensity. This trend is physically consistent with the decreasing trend in the original binary proportion (E/Ec < 1), as both metrics converge on the same conclusion that over recent decades, the physical condition of the water column in coastal regions has become less stable (or more mixed) on average over the study period.

Most importantly, the conclusions remain unchanged. Both stability metrics, the binary proportion and the continuous $\log_{10}$(E/Ec), show the correlations with Chl-a trends remain statistically lower than the correlation between Chl-a and DIP (E/Ec < 1: r = -0.07; $\log_{10}$(E/Ec): r = 0.44; DIP: r = 0.74, Supplementary Fig. 3). This consistent result across two independent measures provides robust, multifaceted evidence against hypothesis H1 that the global trend in physical water-column stability is not the primary driver of the observed multi-decadal increase in coastal phytoplankton biomass. In the revised manuscript, we incorporate the analysis using the continuous $\log_{10}$(E/Ec) as a more comprehensive index to test H1, retaining the original binary proportion analysis to demonstrate the robustness and consistency of our conclusion in terms of frequency and intensity of physical conditions (Lines 114-125 in clean version).

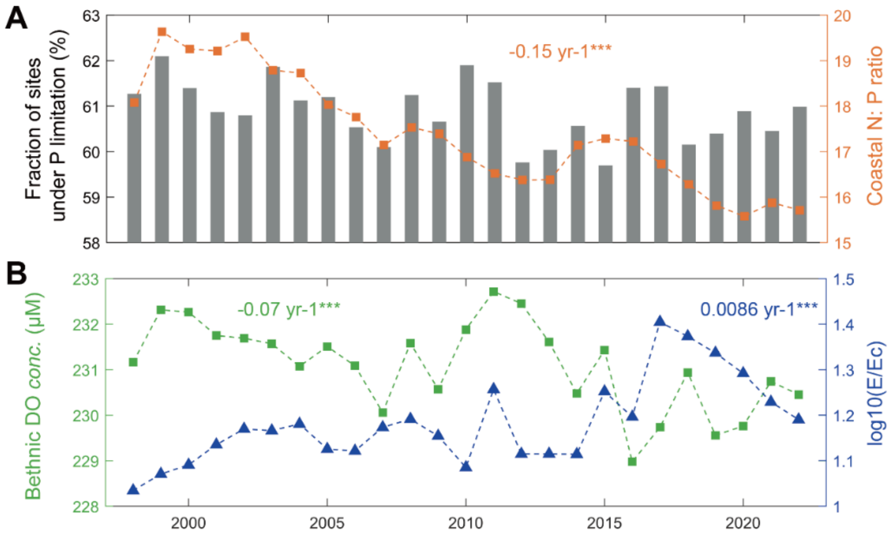

Supplementary Fig. 2. (A) Interannual variability of coastal N: P ratios and the fractions of sites under P limitation (%). (B) Interannual variability of benthic DO concentrations (μM) and $\log_{10}$(E/Ec). The changing rate per year for N: P ratios, DO and log10(E/Ec) is calculated by the ratio of slope to intercept for the linear regression equation. *** indicates the p value < 0.001.

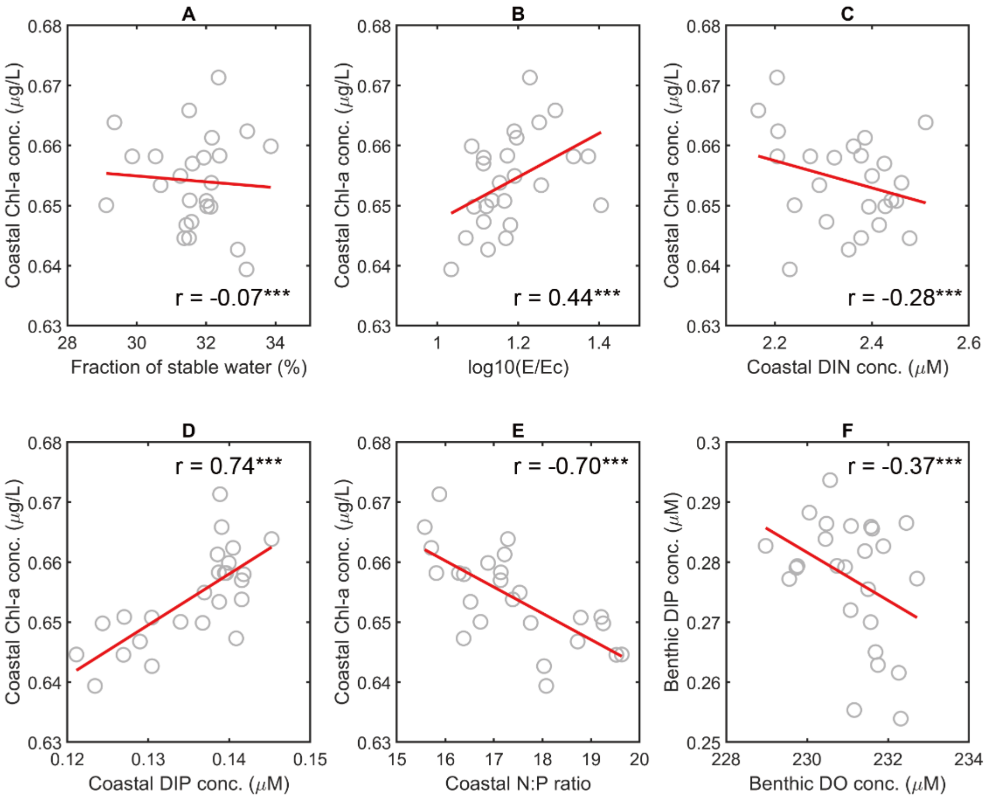

Supplementary Fig. 3. The correlations between annual mean of coastal Chl-a concentrations (μg L^{-1}) and fraction of stable water (%) (A), log_{10}(E/Ec) (B), coastal DIN concentrations (μM) (C), coastal DIP concentrations (μM) (D), coastal N:P ratios (E) and the correlation between benthic DIP concentrations (μM) and benthic DO concentrations (μM) (F), globally. *** indicates the p value < 0.001.

Major comment 4: H2 is considerably less problematic, as it amounts to testing whether changing nutrient and Chl-a concentrations in the same grid cells are correlated. Unsurprisingly, Chl-a concentrations are positively correlated with DIP concentrations, both of which are increasing, and negatively correlated with N:P ratios; DIN concentrations are declining overall, and also negatively correlated with Chl-a. The authors make a compelling argument, based on a large review of the groundwater nutrient/redox literature that DIP is becoming more available in coastal groundwaters due to the increased prevalence of reducing conditions, leading to desorption of sediment-bound P. These same conditions support increased denitrification, leading to declining N concentrations, and even lower N:P in groundwater. I found it a little odd that they didn't seem concerned with testing a hypothesis (H3b?) that increasing hypoxia/anoxia in shallow coastal waters may also be releasing P from sediments. They tend to dismiss such sources as "localized and insufficient to explain the coherent DIP signal (34)", yet the cited reference (Wallman, 2010) notes that "The expansion of oxygen minimum zones [Stramma et al., 2008] may increase the benthic fluxes of phosphate at the continental margins. The further expansion of oxygen minimum zones predicted by ocean modeling [Oschlies et al., 2008] may be amplified by the enhanced benthic phosphate release from margin sediments in a positive feedback loop." It seems that this mechanism, enhanced release of sediment

bound P in shallow coastal waters due to increased hypoxia/anoxia, is also likely to be relevant, and worth investigating using the same approach taken by the authors.

Response: We thank the reviewer for raising this important alternative mechanism. We agree that benthic DIP released by coastal hypoxia is a plausible process. The core of our argument is not that benthic flux is unimportant, but that the coherent, large-scale increase in DIP we observe (Fig. 2B) is more parsimoniously explained by a land-based source (groundwater) responding to large-scale anthropogenic forcing (subsurface redox shifts), rather than by localized sediment processes.

Following the reviewer's suggestion, we used our data to test for a signature of hypoxia-driven benthic release. We directly tested the correlation between bottom dissolved oxygen (DO) at ~26.5 m depth (within our defined coastal zone of <30 m) and coastal DIP concentration across our study grid cells. The correlation is negative ($r = -0.37$, Supplementary Fig. 3F), as expected, but it is substantially weaker than the correlation between groundwater DIP and coastal DIP ($r = 0.70$, Supplementary Fig. 3A). In the revised manuscript (Lines 227-230), we have explicitly addressed the potential role of internal phosphorus loading, specifically, the well-documented bloom-induced hypoxia cascade that triggers benthic DIP release [*Conley et al.*, 2009; *Testa and Kemp*, 2012; *Vahtera et al.*, 2007]. This correlation indicates that while internal benthic P release undoubtedly occurs, and may be exacerbated by global ocean deoxygenation trends [*Oschlies et al.*, 2008; *Stramma et al.*, 2008], it typically acts as a secondary, localized amplifier rather than the primary driver. Consequently, the bloom-hypoxia-benthic DIP cascade is insufficient to explain the robust, large-scale positive correlation observed between DIP and Chl-a in our study. This finding solidifies our conclusion that increased overall external phosphorus availability fundamentally fuels coastal bloom intensification, a conclusion that strongly aligns with recent assessments of global anthropogenic phosphorus imbalances and inputs [*Pei et al.*, 2026; *Wallmann*, 2010].

Comments on the Figures

Figs:

Fig 2: It is worth noting that most of the increase in DIP shown in figure 2 occurs prior to 2010 and then the trend appears to flatten or even decline. Also, figure 2d is not helpful, and somewhat confusing.

Response: We thank the reviewer for these precise observations. The reviewer is correct that the steepest increase in coastal DIP occurred prior to ~2010, with a subsequent flattening. This timing is notable and may correspond to the broader implementation of phosphorus management policies in wastewater treatment and agriculture in many developed regions post-2010, potentially slowing the rate of new phosphorus loading to watersheds. However, the legacy phosphorus stored in soils and aquifers from decades of prior inputs continues to be mobilized and discharged via groundwater, sustaining elevated coastal DIP levels. Thus, the long-term trend (1998-2022) remains significantly positive, which is the key point for testing H2. We agree that the original presentation of two

conceptual models in Fig. 2D was unclear. Its purpose was to illustrate how different initial nutrient input stoichiometries ("High N:P" vs. "Low N:P" source models) lead to distinct system states (DIN-rich vs. DIP-rich) after biological uptake, helping to explain the observed global patterns (declining DIN, rising DIP, declining N:P). To clarify this, we combined original Fig. 2D into Supplementary Fig. 5. This revision maintains the useful conceptual link between the proposed mechanism and the observed nutrient trends while eliminating the previous confusion.

Fig 3d: Rather dense. Is it necessary to combine bar charts and xy plots? I don't see an advantage, so would separate them or plot everything as xy plots. In any case, please explain the subtleties in more detail (arrows, shading, etc)

Response: Thanks for the suggestion. In the revised manuscript, we have replotted the time-series data of two sites for clarity. All subtle features (arrows indicating bloom onset, shading for bloom periods) are well explained in detail in the revised figure caption.

S4: A companion to figure 2d and equally uninformative. These alternative "models" should be explained in a forthright manner in the text. I find them meaningless on their own. If they are included, they should appear as panels a and b to facilitate comparison.

Response: Thanks for the suggestion. We combined them into the Supplementary Fig. 5.

Fig S5a,b: These appear to be frequency histograms of Eh(v), but the text refers to them as histograms of groundwater DIP and DIN "verse groundwater ORP (Eh (V))". I don't understand what this means. Please clarify.

Response: Thanks for pointing out this lack of clarity. We revised the figure caption as "(A) Histogram of field observations of groundwater ORP (Eh (V)) for Fig. 4C. (B) Histogram of field observations of groundwater ORP (Eh (V)) for Fig. 4D.

Fig S7: Far too much information being displayed on the same figures. Please break them into separate panels and explain them in associated text.

Response: We agree with the reviewer that the original Fig. S7 attempted to convey too much information in a single composite figure, compromising clarity. After careful consideration, we have decided to remove this figure entirely from the revised manuscript.

Other comments:

Line 113: "rules out"? Given the resolution of the available data, perhaps "fails to support" is a better choice of words.

Response: Thanks, and we rephased the terminology to moderate the tone.

Lines318-330: It is entirely unclear how this section on global fertilizer use and phosphate rock production relates to the main focus of the study. The authors should be aware of much more thorough studies of global P sources and their environmental consequences. See Pei et al. (2016) for a recent example; there are many others. Such analyses might provide more robust means of estimating P to groundwater and coastal waters.

Response: Thanks for the comments. We agree that the correlation between global P fertilizer and rock production is weak to our main story, therefore after careful consideration, we have decided to remove this discussion entirely from the revised manuscript.

Line 347: Shouldn't it read "E decreases due to damping effects"?

Response: Yes, we have revised.

Lines 350-351: Can the authors point out where there is a reference to E and its empirical constants in (53)?

Response: Sorry for citing wrong, the reference 70 includes the E and the empirical constant. (Munk, W. H. & Anderson, E. R. Notes on a theory of the thermocline. (1948).)

Line 390: diatoms blooms or dinoflagellates blooms -> diatom blooms or dinoflagellate blooms

Response: Thanks, and we revised.

Throughout: The paper has a few misspellings (e.g. "tones" for "tons"), minor grammatical errors, etc throughout the text and supplemental material.

Response: We sincerely thank the reviewer for their careful reading and for pointing out these errors. The entire manuscript and the supplementary material have undergone thorough proofreading to improve overall clarity and consistency.

Reviewer #2 (Remarks to the Author):

This manuscript presents a potentially important and highly original framework linking coastal groundwater phosphorus inputs to the recent intensification of coastal algal blooms (CABs). The study addresses a major scientific question and integrates global nutrient datasets, satellite-derived Chl-a, groundwater geochemistry, and local time-series observations into a coherent hypothesis-driven narrative. In particular, the proposed linkage between coastal aquifer redox conditions, dissolved inorganic phosphorus (DIP) mobilization, and altered coastal nutrient stoichiometry is conceptually novel and could have broad implications for coastal biogeochemistry and eutrophication management. However, I have several major concerns regarding the robustness of the central conclusions.

Response: Thank you for your encouragement. We have made itemized changes to address your comments.

Major comment 1: First, the manuscript relies heavily on satellite-derived Chl-a trends in optically complex coastal waters (<30 m depth), where Chl-a retrieval uncertainties are often substantial due to suspended sediments, CDOM, shallow-water effects, etc. The authors interpret increasing Chl-a as evidence for "global CAB acceleration" but it remains unclear whether the reported trends reflect true phytoplankton biomass increases or, at least partly, retrieval artifacts associated with changing coastal optical properties.

Response: We thank the reviewer for this critical and valid concern regarding data reliability in optically complex coastal zones. We have strengthened our analysis and clarification in response. The core of our long-term trend analysis (supporting H1) utilizes the Copernicus Marine global

biogeochemical reanalysis product GLOBAL_MULTIYEAR_BGC_001_029. This is a model-based hindcast generated by the NEMO3.6-PISCESv2 coupled physical-biogeochemical model, not only a direct satellite retrieval product. The variables chlorophyll-a, nitrate, and phosphate are simulated outputs of this model, which integrates physical dynamics, nutrient inputs, and biogeochemical cycles. Therefore, the generation of these Chl-a fields is fundamentally independent of the optical inversion algorithms that are susceptible to interference from such as suspended sediments and CDOM in coastal waters. This addresses the core of the reviewer's concern about retrieval artifacts. Also, this product has undergone formal quality assessment. As per its Quality Information Document (QUID), it has been validated against satellite ocean color climatology and the World Ocean Atlas 2013 (WOA2013) climatology which is a gridded product based on decades of in-situ measurements. This validation confirms the product's capability to reproduce large-scale spatial patterns and seasonal cycles of biogeochemical variables. While all models have uncertainties, the use of this physics-constrained, globally-consistent reanalysis provides a robust dataset for identifying multi-decadal, large-scale trends, which is the primary goal of our H1 analysis.

To further ensure the reliability of the observed increasing biomass signal, we have implemented additional checks in our revised manuscript. We compared the Chl-a trends from the CMEMS reanalysis with published global bloom frequency[*Dai et al.*, 2023]. The direction and magnitude of the trends showed significant positive correlation ($r = 0.52$, $p < 0.001$, Fig. R1), further confirming the model's trend is not an artifact. The convergence of evidence from a physics-based model (which avoids optical retrieval issues), its agreement with an independent global satellite product, provides strong support that the reported increasing Chl-a trend reflects a real change in coastal phytoplankton biomass.

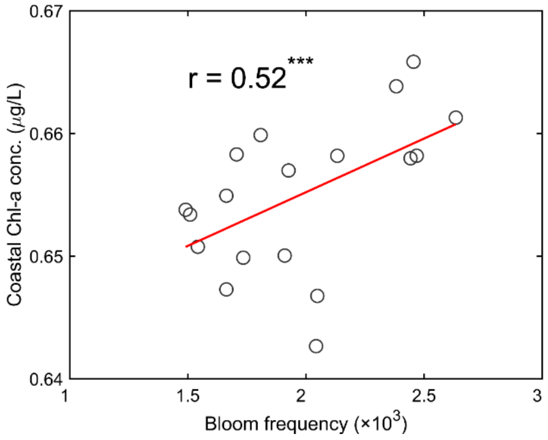

Fig. R1. The correlation between Chl-a concentration from CMEMS reanalysis and published bloom frequency.

Major comment 2: Second, the manuscript may overstate the causal role of groundwater-derived DIP. While the presented correlations strongly suggest that groundwater can act as an important phosphorus source under reducing conditions, the current analyses do not sufficiently constrain the relative contribution of groundwater phosphorus fluxes compared with other major phosphorus

sources such as sediment regeneration, rivers, wastewater, and atmospheric deposition. In particular, sedimentary phosphorus recycling under reducing conditions may produce patterns similar to those attributed here to groundwater inputs. Therefore, the conclusion that groundwater is the dominant driver of recent global CAB acceleration appears stronger than what the available evidence can fully support.

Response: We thank the reviewer for this essential critique, which prompts a more precise interpretation of our evidence. We agree that a definitive, quantitative global flux partitioning between all potential P sources is important but beyond the scope of our correlative and mechanistic study. Our revised argument aims to establish that advective submarine groundwater discharge (SGD) is a major, pervasive, and stoichiometrically congruent driver, rather than claiming precise dominance in flux. We justify this through a multi-faceted analysis that addresses the specific sources mentioned. In the revised manuscript, we provide a detailed discussion of why classical nutrient sources are unlikely to be the primary cause of the observed coherent, low N:P DIP surplus. For example, for inputs from rivers, atmosphere and wastewater, they are predominantly high N:P sources, which would elevate coastal DIN and N:P, contradicting the observed global declines in both (Fig. 2B). For molecular diffusion from sediments, this process is severely limited by scavenging at the oxic sediment-water interface and is too localized to generate the large-scale signal. In contrast, SGD acts as a massive, diffuse "non-point source." Crucially, in reducing coastal aquifers, it functions as a biogeochemical transformer, coupled DIP release from sediments and concurrent denitrification generate and export fluxes with consistently low N:P ratios. This unique stoichiometric signature aligns perfectly with the observed coastal nutrient trends (increasing DIP, decreasing DIN/N:P), making it a parsimonious explanation for the large-scale pattern.

In addition, we directly addressed the reviewer's parallel concern about benthic P release at reducing environments. Analyzing our global dataset, we find the correlation between bottom-water DO and coastal DIP, while negative as expected, is relatively weak ($r = -0.37$, Supplementary Fig. 3F). This is substantially weaker than the groundwater-seawater DIP correlation ($r = 0.70$, Fig. 3A). This, combined with the understanding that sedimentary P release is often localized feedback to prior blooms (and thus cannot be the initial driver of the widespread trend), indicates it is unlikely to be the primary mechanism behind the coherent global DIP increase.

Major comment 3: Finally, I encourage the authors to moderate some of the terminology throughout the manuscript. The study primarily analyzes long-term mean Chl-a trends rather than direct bloom metrics (e.g., bloom frequency, duration, or HAB occurrence records). Thus, terms such as "coastal phytoplankton biomass increase" or "enhanced eutrophic conditions" may be more appropriate than repeatedly referring to "global CAB acceleration." Overall, this is an ambitious and potentially high-impact study, but the manuscript would benefit from a more cautious interpretation of the remote sensing evidence and a clearer assessment of uncertainties and source attribution.

Response: We sincerely thank the reviewer for this precise and constructive suggestion. We agree

that our original terminology could be interpreted as overstating the direct evidence for discrete bloom events. Our model-based analysis indeed only quantifies changes in mean phytoplankton biomass (as Chl-a concentration) over time. We have revised the terminology throughout the manuscript as suggested.

Reviewer #3 (Remarks to the Author):

General comment: This manuscript addresses an important scientific question concerning the continued acceleration of coastal algal blooms under changing nutrient regimes. The authors propose that DIP derived from coastal groundwater represents a previously underappreciated driver of global coastal algal bloom acceleration. The study integrates multiple lines of evidence, including global reanalysis products, a large synthesis of paired groundwater-seawater nutrient observations, site-specific time series, and groundwater redox data. Overall, the manuscript is well structured, and the sequence of hypotheses is logically developed. The proposed mechanism has important implications for coastal nutrient management. Nevertheless, several aspects of the evidence chain and methodology require further clarification and strengthening before the central conclusion can be fully supported.

Response: We thank the reviewer for the thoughtful and constructive feedback, which significantly helps refine our arguments and strengthen the manuscript. We address each major and minor comment below.

Major comments

1. The groundwater evidence should be linked more explicitly to nutrient fluxes rather than concentrations alone. The global groundwater synthesis is based mainly on paired groundwater and adjacent seawater DIP/DIN concentrations. This is valuable, but algal bloom responses depend on nutrient loads or fluxes, not concentrations alone. Groundwater-derived DIP flux depends on both DIP concentration and submarine groundwater discharge rate. Therefore, high groundwater DIP concentration does not necessarily indicate a large contribution to coastal nutrient budgets. Site-level SGD rates or published nutrient flux estimates should be incorporated to demonstrate whether groundwater DIP inputs are quantitatively sufficient to support the observed bloom responses.

Response: This is a critical point. We agree that flux (concentration × discharge) is the ideal and ultimate determinant of ecological impact. However, our use of concentration is a necessary proxy due to the global scarcity of concurrent, co-located DIP concentration and SGD rate measurements. We strengthen the argument as follows. Firstly, by using site-level data in Hong Kong, we have demonstrated the same conclusion from both flux and concentration that groundwater DIP inputs are quantitatively sufficient to support the observed bloom responses. As published as *Cheng et al.* [2024], we calculated DIP loadings by integrating SGD flux and groundwater DIP concentrations and confirmed that high loadings lead the high Chl-a concentrations and algal cell counts, and also we performed a strong correlation ($R^2 = 0.56$) between the groundwater DIP loadings to receiving waters (total DIP inventory) (Fig. R2). We also observed the difference in the magnitude of SGD (~3.1 times) and groundwater DIP concentrations (~14.8 times) (Supplementary Fig. 6A). This

indicates that the groundwater-derived DIP flux mainly depends on DIP concentration rather than SGD rates at this site. To further expand it globally, we made a comparison between archived global groundwater DIP concentrations (Fig. 3A) and global SGD rates. We calculated the global SGD and classified it into 31 marginal seas (the estimation is given in Supplementary notes). We also observed that at the global scale, DIP concentration in coastal groundwater is the first-order control on the potential DIP flux. SGD rates vary, but typically within 1-2 orders of magnitude (0.05-5.56 km3/year), while groundwater DIP concentrations can vary over 2-3 orders of magnitude (0.08-123.2 uM) (Supplementary Fig. 6B). Therefore, concentration is also considered as the dominant variable determining flux magnitude at global scale, so we used the groundwater concentrations to perform our analysis in this study. We accordingly modify the main text (Lines 198-203) and supplement global SGD calculation in 31 marginal seas (Supplementary notes) to justify that we use concentration as a robust indicator of the potential and stoichiometric signature of groundwater inputs, and that where concentration is high, even modest SGD rates can yield significant loads.

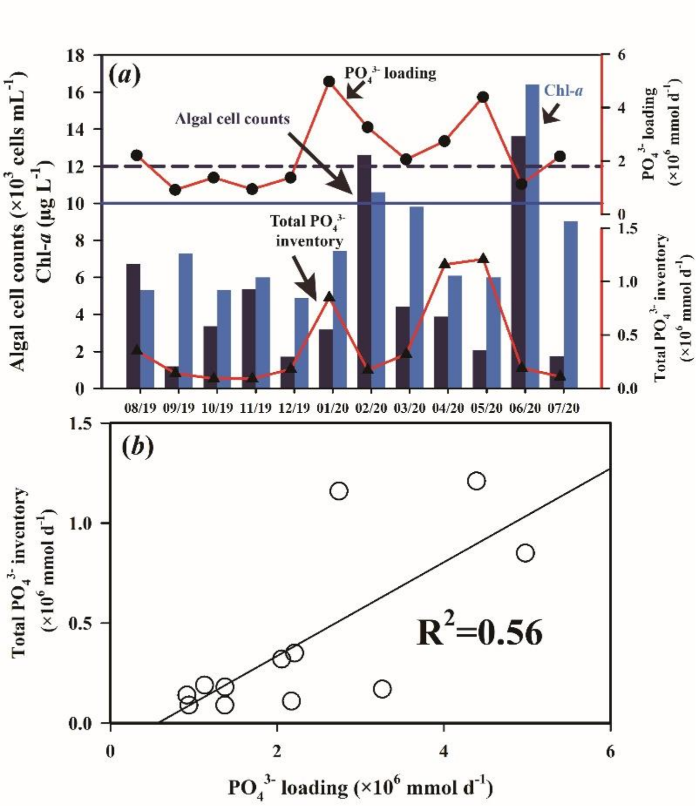

Fig. R2 (a) Monthly distributions of algal cell counts, chlorophyll-a concentrations (Chl-a), groundwater PO_4^{3-} loading and total PO_4^{3-} inventory in seawater. (b) The correlation between the groundwater PO_4^{3-} loading and total PO_4^{3-} inventory in seawater.

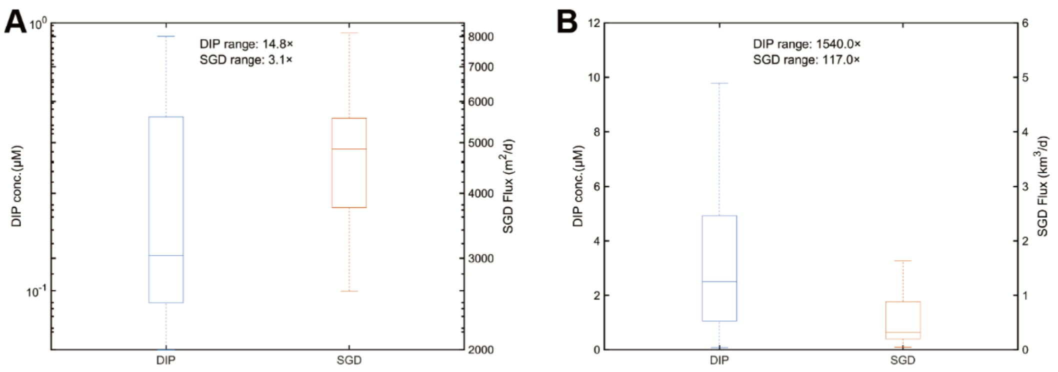

Supplementary Fig. 6. Boxplots of groundwater DIP concentrations and SGD for Hong Kong site (A) and global scale (B). Data for global DIP concentration range is sourced from Fig. 3A, and the estimation of global SGD is given in Supplementary Note.

2. Although DIP–Chl-a co-occurrence is higher than that of DIN or water-column stability, the substantial co-occurrence values for DIN and stability suggest that these factors may still be important controls in many coastal systems. The manuscript would be strengthened by further characterizing the sites where DIN–Chl-a or stability–Chl-a co-occurrence is strong. For example, the authors could examine whether these sites differ in geographic distribution, nutrient limitation regime, hydrodynamic setting, human nutrient inputs, or groundwater redox conditions. This would help clarify where groundwater-derived DIP is likely to be the dominant control and where other mechanisms may be more important.

Response: We thank the reviewer for this excellent suggestion. As noted in our previous responses to **Reviewer 1**, we have carefully moderated our tone throughout the revised manuscript, stepping away from attempts to falsify physical drivers and explicitly acknowledging the fundamental importance of mixing/stability.

To address this comment and better contextualize these alternative mechanisms, we have conducted a detailed spatial analysis of the long-term trends in Chl-a, DIP, DIN, and physical stability (E/Ec) based on Supplementary Fig. S1. This analysis reveals distinct geographical and mechanistic regimes.

- DIP dominance: Significant increasing trends in DIP are overwhelmingly concentrated along densely populated and industrialized coasts (e.g., eastern Asia, North America, and Europe). This spatial pattern perfectly aligns with global hotspots of anthropogenic discharge, robustly supporting our hypothesis that groundwater-derived DIP is the primary driver of widespread water quality deterioration.
- DIN suppression and shifting nutrient limitation: Conversely, DIN exhibits widespread decreasing trends globally. This is consistent with enhanced denitrification under reducing groundwater conditions (H2). Importantly, this decoupling (declining DIN, increasing DIP) implies a global shift in coastal nutrient limitation regimes. As coastal waters transition toward phosphorus-limited or co-limited states, the stimulatory effect of groundwater-derived DIP on Chl-a is significantly amplified.

- Stability dominance (localized physical control): Significant positive trends in water-column stability (increasing stratification) are geographically restricted primarily to semi-enclosed basins (e.g., the Mediterranean Sea, parts of the Gulf of Mexico, and select fjords). In these specific systems, climate-driven physical processes (warming/freshwater input) govern phytoplankton dynamics rather than widespread nutrient loading.

By incorporating this spatially explicit discussion into the revised text (Lines 151-162), we clarify our reframed narrative. While physical stability and DIN dynamics are undeniably crucial controls in localized contexts (e.g., enclosed bays), the large-scale, globally coherent increase in DIP remains the principal baseline driver explaining the bulk of the recent Chl-a intensification.

3. The two site-specific case studies require clearer justification and more direct presentation of site-level N:P evidence. The Hong Kong and Delaware examples provide useful support because groundwater DIP enrichment appears to precede bloom events and is followed by DIN drawdown. However, the manuscript refers to them as “two contrasting sites” without clearly explaining the basis of the contrast. The authors should specify in what sense these two sites are contrasting, such as climate, geomorphology, hydrodynamics, aquifer type, nutrient sources, or bloom characteristics. In addition, the site-specific groundwater N:P ratios should be reported more clearly to show whether low-N:P groundwater input is directly observed before bloom events. In Fig. 3D, the axis scaling or plotted values of groundwater N:P ratio appear unclear and may be inconsistent with the interpretation of low-N:P groundwater input. The authors should check the calculation, axis labeling, and interpretation of groundwater N:P ratios in this figure.

Response: We thank the reviewer for these valuable suggestions. First, our initial aim of using the word of “contrast” was to demonstrate that the groundwater DIP mechanism is not limited to a single coastal type but can operate across diverse environmental settings. To avoid misunderstanding, we have replaced “two contrasting sites” with “two geographically and hydrologically distinct sites”.

Regarding the N:P evidence, we have carefully reviewed and revised the presentation in Fig. 3D-E for clarity. To better visualize the wide range of N:P values and to clarify the timing of low-N:P groundwater input, we have applied a logarithmic transformation to the N:P ratio axes in the revised figures. In Fig. 3D, the groundwater N:P ratio (orange line, right axis) is plotted on a log scale. Before the bloom events (indicated by the grey bars and peaks in Chl-a), there is a distinct period where the groundwater N:P ratio drops to low values accompanying an increased DIP concentration. This precedes the sharp decline in the coastal N:P ratio (blue line), which falls below the Redfield ratio (~16) at the bloom peak. Similarly, in Fig. 3E, the groundwater N:P ratio exhibits low values preceding the seasonal bloom development, again consistent with the interpretation that P-rich (low N:P) groundwater inputs are driving the coastal biogeochemical response. We have revised the axis labels and added annotations to explicitly guide the reader through this interpretation. The calculated Pearson correlation coefficients for the N:P ratios further support the tight coupling between groundwater and coastal N:P dynamics.

4. The linkage between groundwater redox state and bloom trends is promising, but the temporal evidence for changes in groundwater redox conditions remains unclear. The manuscript provides a temporal analysis showing that the effect of groundwater-derived DIP may depend on shifts in coastal nutrient limitation regimes. However, the evidence linking aquifer redox state to CAB trends appears to be based mainly on spatial association among regions with different bloom trends. It remains unclear whether groundwater redox conditions themselves have changed over time in regions where blooms have intensified. The authors should clarify whether reducing aquifers represent a pre-existing vulnerability that enhances bloom responses under phosphorus limitation, or whether temporal changes in groundwater redox conditions have directly contributed to increasingly severe blooms.

Response: We thank the reviewer for this insightful question, which helps clarify the precise role of aquifer redox state and the temporal dimension of our mechanism. The spatial association in Fig. 4 is designed to identify a large-scale, predisposing condition. Aquifers with inherently reducing conditions constitute a widespread vulnerability. This vulnerability becomes ecologically consequential as coastal waters shift towards phosphorus limitation, making them acutely sensitive to groundwater DIP inputs. While multi-decadal trends in aquifer redox at the global scale are not available, we can examine shorter-term, process-based dynamics that underpin the mechanism.

Following the reviewer's suggestion, we present a new analysis of a year-long, high-frequency monitoring dataset from our Hong Kong site (the same site featured in Fig. 3D). This site provides a unique process window into the dynamic sequence linking redox, DIP, and blooms (Fig. 4E). As shown in Fig. 3D, we observed two algal bloom events in February and June 2020 and in the 1-2 months preceding identifiable bloom events, we observed significant increase in groundwater DIP. Fig. 4E shows the iron Pourbaix diagram with the one-year Hong Kong site samples. We separated the data collected in January, April and May 2020 from other months. Our results demonstrate that data from these months preceding identifiable bloom events are mostly located in Fe^{2+} region representing for reducing aquifer environments, accompanying with a higher groundwater DIP concentration (Fig. 3D), while for others, they are mainly located in Fe_2O_3 zone. This high-resolution time-series data provides direct evidence that within a vulnerable (pre-existing reducing) aquifer, short-term fluctuations towards even more reducing conditions can modulate the timing and magnitude of DIP release events, thereby contributing to blooms. Thus, we clarify that at the global/decadal scale, we identify pre-existing aquifer redox state as a key spatial vulnerability factor explaining the pattern of algal bloom increase, and at the local/event scale, our new data analysis shows that dynamic shifts within the reducing aquifer can also modulate the timing and magnitude of DIP release events, thereby contributing to the intensity of individual blooms. Therefore, both aspects are important. We have added the new analysis and results in the revised manuscript (Lines 238-291).

5. The authors resampled the global datasets to 1/24° resolution for consistency among physical, chemical, and biological variables, but the manuscript does not justify whether this spatial resolution

is sufficient to resolve nearshore groundwater discharge signals. Given that SGD and associated nutrient gradients often occur at much smaller spatial scales, the authors should discuss the potential scale mismatch and test whether the results are robust to alternative coastal-zone definitions or spatial resolutions.

Response: We thank the reviewer for raising this important point regarding the potential scale mismatch between our analysis grid (~4 km at 1/24°) and the typical scale of SGD plumes. We acknowledge SGD plumes are often sub-grid scale phenomena, though their influence can extend over considerable distance (up to 40-50 km) in some regions [*George et al.*, 2020; *Michael et al.*, 2016; *Moore and Wilson*, 2005; *Wilson et al.*, 2024]. This resolution was adopted to establish a consistent, global-scale framework for integrating long-term, model-derived trends in physics (water-column stability), chemistry (nutrients), and biology (Chl-a). The goal is to identify emergent, large-scale patterns (e.g., Fig. 2) coherent over decades. At this scale, a grid cell represents the integrated water quality of a coastal area, encompassing signals from various point and non-point sources after lateral mixing. Crucially, in our manuscript, we did not directly correlate these grid-cell values with our groundwater point data. To explore the origin of excess phosphorus, we conducted a ground-truth meta-analysis using a strict criterion that we only used paired groundwater and seawater samples collected in immediate proximity. The global correlations in Fig. 3A-C are based on this paired dataset and illustrate that groundwater DIP concentration is statistically linked to adjacent seawater DIP concentration, irrespective of the larger analysis grid. Therefore, the core finding of a groundwater-seawater DIP linkage is not subject to the grid-scale mismatch concern.

Regarding the representativeness of the model-derived grid data, we acknowledge that the averaged values (e.g., for stability, DIN, DIP, Chl-a) from a defined coastal zone (e.g., all pixels with depth < 30m) capture an integrated signal. To test the sensitivity of our trend analysis (Fig. 2A-B) to the definition of this coastal zone, we conducted a new analysis using only the innermost, shallowest grid cells (depth < 10 m). The results show that the long-term trends for all parameters in the 10m-depth zone are consistent with those from the 30m-depth zone. Furthermore, the parameter values between the two zones are strongly correlated (Chl-a: $r = 0.62$; DIN: $r = 0.95$; DIP: $r = 0.97$; stability: $r = 0.84$, Fig. R3). This confirms that the key trends reported in our study are robust to alternative coastal-zone definitions.

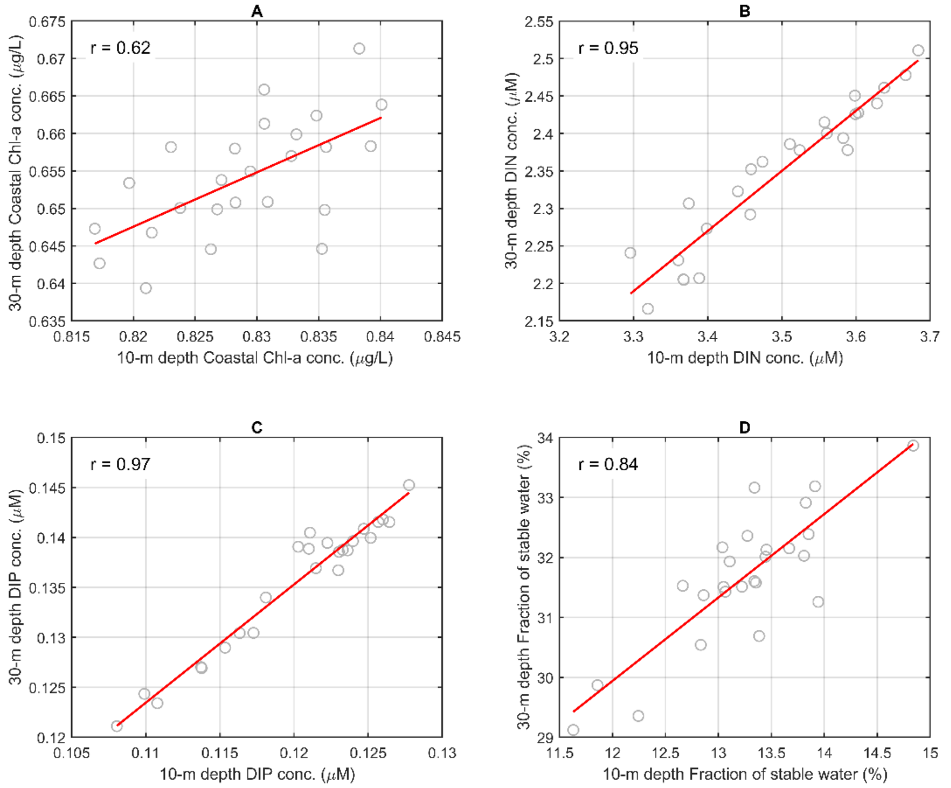

Fig. R3 The correlations of four parameters (Chl-a, DIP, DIN and water stability) between 10-m water depth coastal zones and 30-m water depth coastal zones.

Minor comments

1. The sentence "As coastal populations expand and pressures from resource use intensify…, the urgency to understand and mitigate CABs…" is grammatically incomplete because it lacks a main verb. It could be revised as: "As coastal populations expand and pressures from resource use intensify, it is increasingly urgent to understand and mitigate CABs, not only to safeguard ecosystem resilience but also to protect livelihoods in a changing climate."

Response: Thanks, and we rephrase the sentence.

2. The phrase "two contrasting sites" should be clarified or replaced with a more specific description, such as "two geographically and hydrologically distinct sites," if the contrast is explicitly supported.

Response: Thanks for the comment. We have replaced with "two geographically and hydrologically distinct sites" as suggested**.**

3. The bloom thresholds used for Hong Kong and Delaware differ. The authors should briefly justify these thresholds and indicate whether the results are sensitive to threshold choice.

Response: We thank the reviewer for this point. The site-specific bloom thresholds (Hong Kong: 10 μg L^{-1} (*Cheng et al.* [2024]); Delaware: 20 μg L^{-1} (USEPA (2021)) are based on local, long-term observational data, historical bloom records, and regional water quality guidelines. These thresholds

are empirically defined to represent significant biomass levels indicative of bloom conditions within the specific ecological and hydrodynamic context of each site.

4. It's better to provide more details on the selected 32 coastal regions used for the groundwater redox analysis, preferably in a supplementary table, including location, sample size, redox category, bloom trend category, and data source.

Response: Yes, we provide the supplementary table as suggested (Supplementary Data 2).

5. It should be clarified whether seasonal variability was considered in the groundwater–seawater nutrient comparison. Groundwater nutrient concentrations and coastal blooms can both vary strongly by season.

Response: We thank the reviewer for raising this important point. Seasonal variability was appropriately considered in our analyses, and the approach differs between the global and site scales. For the global-scale correlation analysis (Fig. 3A-C), our paired groundwater-seawater nutrient dataset is a meta-analysis synthesis that aggregates samples collected across different seasons and years from numerous studies globally. By integrating data that inherently captures seasonal and inter-annual variability, the resulting correlations are based on the long-term, aggregate geochemical relationship between the aquifer and adjacent coastal waters. This approach means that the reported strong correlations (e.g., $r = 0.70$ for DIP) reflect a robust, underlying association that is not an artifact of or limited to any specific season. For the site-scale time-series evidence (Fig. 3D-E), the full, high-frequency (e.g., monthly) data are presented. This allows the visualization of seasonal and event-scale dynamics, which is crucial for establishing the mechanistic sequence. The figures clearly show that pulses of high groundwater DIP precede the seasonal development of coastal blooms, providing direct evidence that is consistent with, and not confounded by, seasonal co-variation.

6. The authors should more clearly distinguish between groundwater nutrient concentration and groundwater nutrient flux throughout the manuscript.

Response: We thank the reviewer for this important suggestion to improve the precision of our terminology. In the revised manuscript, we made a clear and consistent distinction.

7. Figure S5 does include groundwater N:P information, but the main text should refer to it more explicitly when discussing low-N:P groundwater inputs.

Response: Thanks, and we have cited the Fig. S5 in the main text clearly.

8. It is not clear whether river-dominated estuaries, enclosed bays, lagoons, and open coasts were analyzed together or separately. These systems have very different hydrodynamic and nutrient cycling characteristics, which may influence the DIP–Chl-a relationship.

Response: We thank the reviewer for this question. In our initial global-scale analysis, all coastal grid cells (defined as areas with ocean depth < 30 m) were analyzed together as an aggregate to identify the overarching, large-scale trend and correlation. We acknowledge that this approach may blend the distinct characteristics of different system types. To address this concern, we have conducted a new sub-group analysis based on a global coastal typology dataset. We show three

major categories, including river-dominated estuaries (Amazon river, Pearl river), enclosed bays (Gulf of Mexico, Bay of Bengal), and open coasts (Eastern coasts of US, Guinea coastal line). Our results indicate that the positive correlations between DIP and Chl-a remain statistically significant across all these major coastal types, although the strength of the correlation varies (r = 0.34-0.87, Supplementary Fig. 4). This suggests that the reported DIP-Chl-a relationship is a robust feature not driven solely by one specific system type.

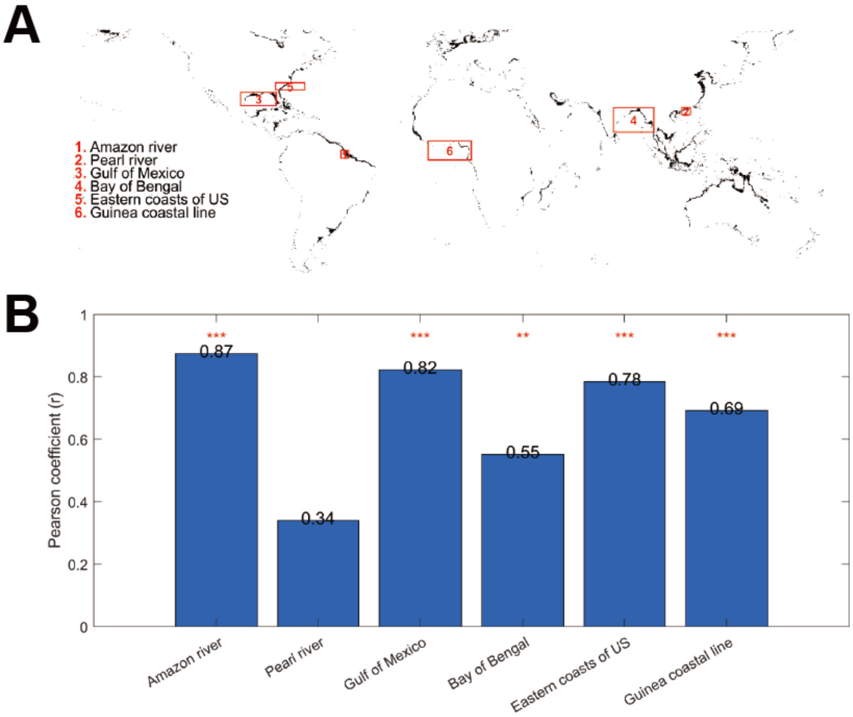

Supplementary Fig. 4. Illustration of the Pearson coefficient (r) between coastal DIP concentrations and coastal Chl-a concentrations based different coastal systems (river-dominated estuaries (Amazon river, Pearl river), enclosed bays (Gulf of Mexico, Bay of Bengal), and open coasts (Eastern coasts of US, Guinea coastal line)).

9. The authors should also clarify whether the groundwater-seawater paired nutrient database includes both fresh groundwater discharge and recirculated seawater through subterranean estuaries. These two pathways may have different nutrient signatures and management implications.

Response: Our paired groundwater and nutrient database predominantly consist of recirculated seawater. We have explicitly clarified this in the revised Methods section (Lines 350–358).

Reference

Cheng, K., J. J. Jiao, J. H. Lee, and X. Luo (2024), Synergistic controls of water column stability and groundwater phosphate on coastal algal blooms, *Water Research*, *255*, 121467.

Conley, D. J., S. Björck, E. Bonsdorff, J. Carstensen, G. Destouni, B. G. Gustafsson, S. Hietanen, M. Kortekaas, H. Kuosa, and H. Markus Meier (2009), Hypoxia-related processes in the Baltic Sea, *Environmental science & technology*, *43*(10), 3412-3420.

Dai, Y., S. Yang, D. Zhao, C. Hu, W. Xu, D. M. Anderson, Y. Li, X.-P. Song, D. G. Boyce, and L. Gibson (2023), Coastal phytoplankton blooms expand and intensify in the 21st century, *Nature*, *615*(7951), 280-284.

George, C., W. S. Moore, S. M. White, E. Smoak, S. B. Joye, A. Leier, and A. M. Wilson (2020), A new mechanism for submarine groundwater discharge from continental shelves, *Water Resources Research*, *56*(11), e2019WR026866.

Michael, H. A., K. C. Scott, M. Koneshloo, X. Yu, M. R. Khan, and K. Li (2016), Geologic influence on groundwater salinity drives large seawater circulation through the continental shelf, *Geophysical Research Letters*, *43*(20), 10,782-710,791.

Moore, W. S., and A. M. Wilson (2005), Advective flow through the upper continental shelf driven by storms, buoyancy, and submarine groundwater discharge, *Earth and Planetary Science Letters*, *235*(3-4), 564-576.

Oschlies, A., K. G. Schulz, U. Riebesell, and A. Schmittner (2008), Simulated 21st century's increase in oceanic suboxia by CO2-enhanced biotic carbon export, *Global Biogeochemical Cycles*, *22*(4).

Pei, W., H. Zhao, J. Han, C. Sonne, M. Whelan, S. S. Lam, H. Guo, Z. Fan, and M. Li (2026), Global Anthropogenic Phosphorus Inputs and Future Sustainability Solutions, *Environmental Science & Technology*.

Stramma, L., G. C. Johnson, J. Sprintall, and V. Mohrholz (2008), Expanding oxygen-minimum zones in the tropical oceans, *science*, *320*(5876), 655-658.

Testa, J. M., and W. M. Kemp (2012), Hypoxia-induced shifts in nitrogen and phosphorus cycling in Chesapeake Bay, *Limnology and Oceanography*, *57*(3), 835-850.

U. S. E. P. A. (USEPA) (2021) National coastal condition assessment. Waste and cleanup risk assessment.

Vahtera, E., D. J. Conley, B. G. Gustafsson, H. Kuosa, H. Pitkänen, O. P. Savchuk, T. Tamminen, M. Viitasalo, M. Voss, and N. Wasmund (2007), Internal ecosystem feedbacks enhance nitrogen-fixing cyanobacteria blooms and complicate management in the Baltic Sea, *AMBIO: A journal of the Human Environment*, *36*(2), 186-194.

Wallmann, K. (2010), Phosphorus imbalance in the global ocean?, *Global Biogeochemical Cycles*, *24*(4).

Wilson, A. M., A. Osborne, and S. M. White (2024), Large-scale groundwater flow and sedimentary diagenesis in continental shelves influence marine chemical budgets, *Nature Communications*, *15*(1), 1143.

REVIEWERS' COMMENTS

Reviewer #1 (Remarks to the Author):

The authors have adequately addressed the concerns I raised about the previous version of the paper, and I believe it to be a solid contribution to the literature.

Reviewer #2 (Remarks to the Author):

The authors have addressed my comments well, I don't have any further questions.

Reviewer #3 (Remarks to the Author):

My comments have been well addressed.

Response: We thank all the referees for their valuable comments and insightful feedback during the review process that helped us refine our manuscript.
